# Economic burden of in-hospital AKI: a one-year analysis of the nationwide French hospital discharge database

**DOI:** 10.1186/s12882-023-03396-8

**Published:** 2023-11-21

**Authors:** Céline Monard, Thomas Rimmelé, Esther Blanc, Mélanie Goguillot, Stève Bénard, Julien Textoris

**Affiliations:** 1 Service d’Anesthésie-Réanimation, Hôpital Edouard Herriot, Hospices Civils de Lyon, Lyon, France; 2 EA 7426, PI3 (Pathophysiology of Injury-Induced Immunosuppression), Université Claude Bernard Lyon 1, Biomérieux, Hospices Civils de Lyon, Lyon, France; 3 SA, Global Medical Affairs, bioMerieux, Marcy-L’Étoile, France; 4 stève consultants, Oullins, France

**Keywords:** Acute Kidney Injury, Costs, Economic burden, Epidemiology, Nationwide database

## Abstract

**Background:**

Although Acute Kidney Injury (AKI) incidence is increasing worldwide, data investigating its cost are lacking. This population-wide study aimed to describe the characteristics and costs of hospital stays with, and without AKI, and to estimate the AKI-associated increases in costs and length of stay (LOS) in three subgroups (major open visceral surgery (MOV), cardiovascular surgery with extracorporeal circulation (CVEC), and sepsis).

**Methods:**

All hospital stays that occurred in France in 2018 were included. Stay and patient characteristics were collected in the French hospital discharge database and described. Medical conditions were identified using the 10^th^ International Classification of Diseases and the medical acts classification. In each subgroup, the adjusted increase in cost and LOS associated with AKI was estimated using a generalized linear model with gamma distribution and a log link function.

**Results:**

26,917,832 hospital stays, of which 415,067 (1.5%) with AKI, were included. AKI was associated with 83,553 (19.8%), 7,165 (17.9%), and 15,387 (9.2%) of the stays with sepsis, CVEC, and MOV, respectively. Compared to stays without AKI, stays with AKI were more expensive (median [IQR] €4,719[€2,963-€7782] *vs.* €735[€383-€1,805]) and longer (median [IQR] 9[4–16] *vs.* 0[0–2] days). AKI was associated with a mean [95%CI] increase in hospitalization cost of 70% [69;72], 48% [45;50], and 68% [65;70] in the sepsis, CVEC, and MOV groups respectively, after adjustment.

**Conclusion:**

This study confirms the major economic burden of in-hospital AKI in a developed country. Interventions to prevent AKI are urgently needed and their cost should be balanced with AKI-related costs.

**Supplementary Information:**

The online version contains supplementary material available at 10.1186/s12882-023-03396-8.

## Introduction

Acute Kidney Injury (AKI) may concern 1.6% to 20% of hospitalized patients, depending on the methodology used and the geographical area concerned [1, 2]. This incidence is increasing as the population is aging and as the number of medical procedures performed on comorbid patients is becoming more important [3]. At the same time, no new treatment has been made available, and AKI may ultimately require renal replacement therapy (RRT) for the most severe cases, which concerns up to 7% of critically ill patients [4]. Thus, the incidence of AKI requiring RRT is also increasing; a mean 10% increase per year in the United States between 2000 and 2009 has been reported and increasing rates have also been reported more recently in European countries [5–7]. In addition to the high costs driven by RRT [8], the fact that AKI impacts other organ functions may lead to more diagnostic and therapeutic workups [9]. Altogether, AKI may increase healthcare resource utilization and length of stay (LOS), increasing direct hospital costs over the years [10]. Few studies including large and unselected nationwide populations, confirmed the major costs associated with AKI; up to $1.8 billion USD in England and $24 billion USD in the United States, each year [11, 12]. To reduce AKI impact, a particular attention should be paid to populations with a high incidence of AKI, such as sepsis and surgical patients [13, 14], and/or for which preventive measures could be implemented. Thus, nephroprotective bundles have been found to decrease AKI incidence in post-surgical patients [15–17]. The cost of such bundles should be weighed against the cost of AKI itself, which has been shown to be associated with significant cost increases in surgical patients [18, 19]. However, these results are old, calling for updated data. Concerning sepsis, although it is known to be a leading cause of AKI, no study has specifically focused on the cost of AKI in this particular population.

The aim of the present study was to describe in a large, recent, and unselected population, the characteristics and costs of hospital stays with AKI (AKI stays) and those without (no-AKI stays), and to estimate the increase in stays’ costs from the national insurance perspective and LOS, associated with AKI in three subgroups of interest: major open visceral surgery (MOV), cardiovascular surgery with extracorporeal circulation (CVEC), and sepsis.

## Methods

### Study design and ethics

This study is a retrospective cross-sectional population-wide database study, using the French hospital discharge database (*Programme de Médicalisation des Supports d’Information*, PMSI). PMSI is hosted and controlled by the technical agency for information on hospital care (*Agence Technique de l’Information sur l’Hospitalisation*, ATIH). According to French law, data were collected via the platform of the secure data access center (*Centre d’Accès Sécurisé aux Données*, CASD) after a declaration to the national institute of health data (*Institut National des Données de Santé*, INDS) through the reference methodology 006 (MR-006), n°2214295. Because all patient-level data in the PMSI database are anonymized, institutional review board, ethics approval, and informed consent at an individual patient level were not required.

### Data source

The PMSI contains a discharge summary for each single stay that occurs in each public or private hospital in France (approximately 3,000 hospitals in 2018). Each stay is identified by a main diagnosis (the reason for hospital admission) and several associated diagnoses (medical comorbidities of the patient or associated medical conditions and complications that occurred during the same hospital stay). All diagnoses are registered in the PMSI for billing purposes, according to the International Classification of Diseases, 10^th^ revision (ICD-10) codes. The PMSI also contains all medical procedures performed during the stay, encoded according to the French medical classification of clinical procedures (*Classification Commune des Actes Médicaux*, CCAM).

According to the codes entered in the hospital stay resume (including codes for AKI), each stay is classified within a Diagnosis Related Group (DRG) of patients that shares common characteristics and have similar resources use. This DRG code is used to determine the reimbursement of the stay by the national health insurance system to the hospital. For each DRG, two tariffs coexist according to the hospital status (private or public). Supplementary fees are added to the DRG for particular procedures such as RRT, particular stays such as ICU stays and costly treatments.

### Study population

All hospital stays registered in the PMSI between January 1^st^, 2018 and December 31^st^, 2018 were included. AKI stays were identified by the presence of at least one of the following ICD-10 codes (as main, related, or associated diagnosis): acute kidney failure (N17*), post-procedural acute kidney failure (N990), post-partum acute kidney failure (O904), renal failure following ectopic and molar pregnancy (O084), and extrarenal uremia (R39.2). The following codes for RRT related to AKI management were also used to identify AKI patients: JVJF002, JVJF005, JVJF006, JVJF007, JVJB002. A single patient may have experienced several hospitals stays during the same year and could therefore be included more than once in the present study.

Three subgroups of stays associated with medical procedures or conditions at risk for AKI were defined a priori. The stays with MOV and those with CVEC were identified using the corresponding CCAM codes (Supplementary Table 1a). Stays with sepsis were identified using explicit ICD-10 codes for sepsis, as previously described, and following the recommendations issued by ATIH on coding practices that were in force in 2018 ([20–22], Supplementary Table 1b). These groups were not mutually exclusive, a stay with a surgery and a sepsis code was included in both subpopulations.

### Characteristics and cost of stays

Each stay was described using administrative, medical, and demographic characteristics. The administrative characteristics collected were hospital status (private/public), geographical area of residence, origin of admission (from home, medical, surgical, and obstetrical units (MSO) from a different hospital or emergency room (ER)), discharge destination (to home, MSO from a different hospital or rehabilitation care), and LOS. The medical characteristics collected were the main diagnosis, the medical procedures performed during the stay, the treatments received during the stay (vasopressors, mechanical ventilation, oxygen delivery, RRT for AKI), and death during the stay. Patient characteristics such as age and sex were also collected. Because they are the main comorbidities associated with AKI (susceptibilities), three medical conditions were collected using the corresponding CCAM and ICD-10 codes: cardiac failure, diabetes, chronic kidney disease (Supplementary Table 1c, [1, 23]).

The cost related to each stay was estimated from the perspective of the national health insurance system (reimbursement for the stay by the national health insurance to the hospital according to the PMSI data). For each stay, the associated DRG (eventually accounting for the presence of an AKI code) and the corresponding tariff were collected. Additional reimbursements that occurred in case of an ICU stay, medical procedures such as RRT, or costly treatments were also collected and included in the total cost of stay.

### Statistical analysis

The statistical unit for analysis was the hospital stay. The characteristics of AKI and no-AKI stays were described using mean and standard deviation (SD) or median and interquartile range (IQR) for continuous variables, and frequencies and percentages for categorical variables. Because the whole population was included without sampling, statistical tests were not relevant to compare populations. Missing data were not replaced.

To estimate the increase in cost and LOS associated with AKI in the three subgroups, a case–control analysis was performed. Control stays (no-AKI stays) were matched to AKI stays with a 1:3 ratio (i.e., one AKI stay was matched to 3 no-AKI stays in each subgroup). Matching was considered acceptable beyond 90% of patients matched. If less than 90% of patients were matched, a lower ratio was used (1:2 then 1:1). For each AKI stay, control stays were selected randomly among the no-AKI stays sharing the same age class, sex, geographical area of residence, hospital status, and medical condition associated (sepsis for MVO and CVEC, surgery for sepsis group). Two different stays for a single patient could not be paired together. Characteristics of AKI stays and no-AKI stays were compared within each subgroup, using a Student’s *t*-test or a Wilcoxon-Mann–Whitney test for continuous variables depending on the distribution and a McNemar test for categorical variables. Within matched populations, the effect of AKI on the cost of stay and LOS was estimated using a generalized linear model with a gamma distribution and a log link function. The model provided estimates for mean (95%CI) increase in cost and LOS in AKI stays, adjusted for chronic kidney disease, diabetes, and cardiac failure.

## Results

### Study population

**Fig. 1:**
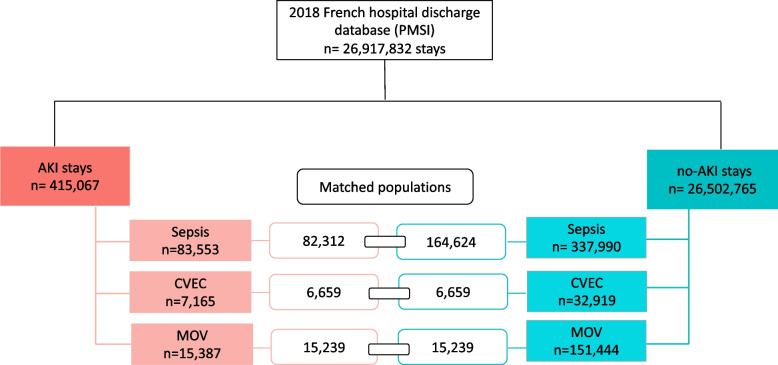
Study flow chart: In the matched populations, cases (AKI stays) and controls (no-AKI stays) were paired based on age class, sex, area of residence, hospital type (public/private), presence of a sepsis (in the surgical subgroups), presence of a surgery (in the sepsis subgroup). *AKI*  Acute kidney injury, *CVEC*  Cardiovascular surgery with extracorporeal circulation, *MOV*  Major open visceral surgery, PMSI  Programme de Médicalisation des Supports d’Information

The 2018 PMSI database contains 26,917,832 stays, of which 9,458,453 (35.1%) were admissions for specific procedures and health care (including radiotherapy and chemotherapy sessions, rehabilitation, and dialysis sessions). All of these PMSI stays were included in the main analysis and a code for AKI was present in 415,067 stays (1.54%). An explicit code for sepsis was present in 421,543 (1.6%) stays, a code for CVEC in 40,084 (0.1%) stays, and a code for MOV in 166,831 (0.6%) stays. An AKI code was associated with 83,553 (19.8%), 7,165 (17.9%), and 15,387 (9.2%) of the sepsis, CVEC, and MOV stays respectively. The study population flow-chart is reported in Fig. 1.

### Characteristics of AKI and no-AKI stays

Public hospitals accounted for 67.7% of the total stays in 2018 (*n* = 18,212,482), and 87.7% of the AKI stays (*n* = 363,988). When compared to no-AKI stays, AKI stays concerned more often male patients (*n* = 235,554, 56.8% *vs. n* = 12,657,336, 47.8%) and older patients (mean [SD] age 74.2[16.4] *vs.* 55.8[23.9]). AKI stays were mainly admitted from the ER (*n* = 227,377, 54.8%), whereas no-AKI stays were mainly admitted from home (*n* = 21,580,666, 81.4%). Discharge was more frequent to home for no-AKI stays (*n* = 24,726,121, 93.3%) than for AKI stays (n = 238,392, 57.4%). Death occurred in 59,338 patients with AKI during their hospital stay (14.3%) and in 237,673 patients without AKI (0.9%; Table 1). The most common DRG in AKI stays were “heart and heart valves diseases, with complications” (*n* = 31,389, 7.6%), “respiratory tract infections, with complications” (*n* = 27,994, 6.7%) and “other kidney and urinary tract diseases, with complications” (*n* = 24,653, 5.9%).

**Table 1:** Characteristics of the population

	**AKI stays (*N* = 415,067)**	**No-AKI stays (*N* = 26,502,765)**	**Whole population (*N* = 26,917,832)**
**Hospital status**			
Private	51,079 (12.3)	8,654,271 (32.7)	8,705,350 (32.3)
Public	363,988 (87.7)	17,848,494 (67.3)	18,212,482 (67.7)
**Stay characteristics**			
Admission			
-From home	136,269 (32.8)	21,580,666 (81.4)	21,716,935 (80.7)
-From another hospital MSO	42,531 (10.2)	465,526 (1.8)	508,057 (1.9)
-From ER	227,377(54.8)	4,296,995 (16.2)	4,524,372 (16.8)
Discharge			
-To home	238,392 (57.4)	24,726,121 (93.3)	24,964,513 (92.7)
-To another hospital MSO	50,507 (12.2)	644,588 (2.4)	695,095 (2.6)
-To rehabilitation care	50,215 (12.1)	618,619 (2.3)	668,834 (2.5)
Main diagnosis at admission^a^			
-Other forms of heart diseases	60,736 (14.6)	877,232 (3.3)	937,968 (3.4)
-Renal failure (including AKI)	51,564 (12.5)	132,141 (0.5)	183,705 (0.7)
-Persons encountering health services for specific procedures and health care	30,010 (7.2)	9,428,443 (35.6)	9,458,453 (35.1)
-Influenza and pneumonia	20,781 (5)	210,870 (0.8)	231,651 (0.9)
Risk factors			
-Sepsis	83,553 (20.1)	337,990 (1.3)	421,543 (1.6)
-Surgery	75,797 (18.3)	7,450,610 (28.1)	7,526,407 (28.0)
-Heart failure	96,673(23.3)	514,199 (1.9)	610,872 (2.3)
-Diabetes	109,004 (26.3)	1,339,975 (5.1)	1,448,979 (5.4)
-Chronic kidney disease	111,746 (26.9)	3,238,058 (12.2)	3,349,804 (12.4)
Treatments during hospital stay			
-Vasopressors	49,853 (12)	77,912 (0.3)	127,765 (0.5)
-Mechanical ventilation	44,900 (10.8)	121,258 (0.5)	166,158 (0.6)
-RRT for AKI	40,436 (9.7)	0 (0.0)	40,436 (0.2)
-Oxygen delivery	57 028 (13.7)	328 014 (1.2)	385 042 (1.4)
Death during stay	59,338 (14.3)	237,673 (0.9)	297,011 (1.1)
Length of stay, mean (SD), days	12.5 (14.7)	2.3 (6.1)	2.5 (6.4)
ICU admission during stay (yes)	71,277 (17.2)	208,265 (0.8)	279,542 (1.0)
-Length of ICU stay, mean (SD), days	19.4 (22.2)	10.1 (13.9)	11.4 (15.6)
**Patient characteristics**			
Age, mean (SD), years	74.2 (16.4)	55.8 (23.9)	56.0 (23.9)
Male sex	235,554 (56.8)	12,657,336 (47.8)	12,892,890 (47.9)

Results are expressed as number (percentage), unless specified

^a^The four most represented categories of ICD-10 codes among AKI-stays

*MSO* Medical Surgical and Obstetrical Units, *ER* Emergency Room, *MV* Mechanical Ventilation, *ECC* Extracorporeal Circulation, *AKI* Acute Kidney Injury, *RRT* Renal Replacement Therapy, *ICU* Intensive Care Unit (excluding intermediate care units), *SD* Standard Deviation

Surgical stays (CVEC and MOV) with AKI required more often RRT than stays with sepsis and AKI (CVEC: *n* = 2,121, 29.6%; MOV: *n* = 4,013, 26.1%; sepsis: *n* = 14.522, 17.4%). However, death occurred more frequently in the sepsis and AKI stays than in the surgery and AKI stays (sepsis: *n* = 24,672, 29.5%; MOV: n = 3,870, 25.2%; CVEC: *n* = 1,196, 16.7%). The characteristics for each subgroup of stays are presented in the Supplementary Table 2.

### Costs and lengths of stay in the whole population

The total cost of stay and its main components are shown in Table 2 for the whole PMSI population and subpopulations. AKI stays were associated with higher costs than no-AKI stays (median [IQR] €4,719.9 [2,963.6–7781.8] *vs* €735.5 [382.8–1805.4]). Patients with AKI required more medical procedures (such as mechanical ventilation, vasopressor infusion and oxygen delivery) than those without AKI (Table 1). Among stays with AKI, 9.7% (*n* = 40,436) required RRT during their stay and 17.2% (*n* = 71,277) had at least one admission to the ICU whereas only 0.8% (*n* = 208,265) of those without AKI were admitted to the ICU. The additional cost of an ICU stay was higher for AKI-stays compare to no-AKI stays (median [IQR] €4,024 [€1,608—€8,844] *vs.* €1,608 [€0- €4,824]).

**Table 2:** Cost of stay (in euros) in different populations

	**AKI stays**	**No-AKI stays**	**Whole population**
**Whole population**			
Number of stays	415,067	26,502,765	26,917,832
Total cost of stay	7,689.2 (11,842.6)	1,630.3 (2,918.3)	1,724.7 (3,336.4)
DRG tariff	5,820.5 (6,387.6)	1,422.9 (2,271.9)	1,491.5 (2,452.2)
ICU supplement	1,338.8 (5,738.0)	35.3 (885.7)	55.7 (1,145.1)
Costly therapies	208.0 (2,127.7)	133.5 (835.7)	134.6 (870.7)
**Sepsis population**			
Number of stays	85,553	337,990	421,543
Total cost of stay	14,882.2 (20,209.5)	7,920.0 (11,314.5)	9,299.9 (13,831.1)
DRG tariff	9,374.3 (9,684.9)	6,367.2 (6,612.8)	6,963.2 (7,422.3)
ICU supplement	4,376.8 (10,783.1)	959.5 (5,377.1)	1,636.9 (6,934.3)
Costly therapies	533.3 (3,721.0)	178.5 (2,112.4)	248.8 (2,518.3)
**CVEC population**			
Number of stays	7,165	32,919	40,084
Total cost of stay	34,288.6 (27,870.7)	18,387.9 (9,626.1)	21,229.9 (15,875.6)
DRG tariff	22,847.5 (13,668.5)	15,266.9 (6,244.9)	16,621.8 (8,593.7)
ICU supplement	8,936.6 (13,985.6)	2,204.6 (3,967.9)	3,407.8 (7,385.0)
Costly therapies	1,328.8 (4,225.7)	243.8 (1,128.8)	437.7 (2,100.1)
**MOV population**			
Number of stays	15,387	151,444	166,831
Total cost of stay	25,476.6 (25,130.6)	9,402.7 (8,738.0)	10,885.1 (12,213.9)
DRG tariff	16,564.0 (11,559.7)	8,536.0 (5,905.2)	9,276.3 (7,026.6)
ICU supplement	6,826.0 (13,813.9)	528.9 (3,739.6)	1,109.6 (5,797.6)
Costly therapies	1,246.8 (5,054.9)	104.3 (1,237.8)	209.7 (1,963.8)

Costs are expressed in mean (standard deviation), euros

*AKI* Acute Kidney Injury, *DRG* Diagnosis related group, *ICU* Intensive Care Unit (excluding intermediate care units), *CVEC* Cardiovascular surgery with extracorporeal circulation, *MOV* Major open visceral surgery

Stays with AKI were longer (median [IQR] 9 [4-16] days *vs.* 0 [0–2] days); 56% (*n* = 232,368) of the AKI stays lasted > 8 days whereas 75.7% (*n* = 20,065,935) of the no-AKI stays lasted < 2 days and 8.2% (*n* = 2,161,042) lasted > 8 days.

### Increase in costs and LOS associated with AKI in the matched subgroups

The case–control study included 82,312 AKI stays with sepsis, 6,659 AKI stays with CVEC, and 15,239 AKI stays with MOV. The pairing ratio was 1:2 in the sepsis group and 1:1 in the surgical groups. As decided a priori, analyses were adjusted based on the presence of chronic kidney disease, diabetes, and cardiac failure.

**Fig. 2:**
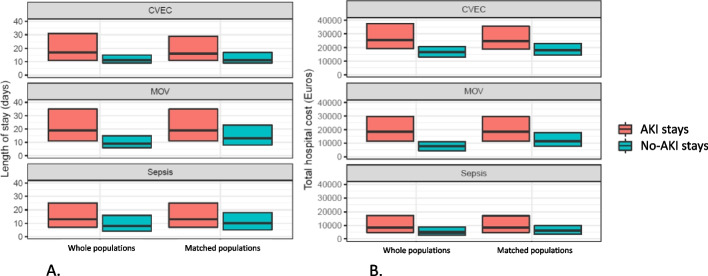
Median length of stays and costs with interquartile range in the whole PMSI population and the matched populations for each subgroup. **A** length of stay in days, median with interquartile range. **B** total hospital cost of stay in euros, median with interquartile range. *p* < 0.0001 for all comparisons between AKI and no-AKI stays in the matched populations, Wilcoxon tests *CVEC*  Cardiovascular surgery with extracorporeal circulation, *MOV*  Major open visceral surgery

 In the sepsis group, compared to the matched no-AKI stays, the presence of AKI increased the hospital cost by 1.70 times (95%CI [1.69; 1.72]; crude median cost of stay [IQR] €8.274,0 [4.769,0–17.076,4] for stays with AKI *vs.* €5.988,5 [3.522,3–9.688,7] for matched stays with no AKI, *p* < 0.0001) and the LOS by 1.33 times (95%CI [1.32; 1.34]; crude median LOS [IQR] 13 [7-25] days for stays with AKI *vs*. 10 [5-18] days for matched stays with no AKI, *p* < 0.0001). In the CVEC group, the presence of AKI increased the hospital cost by 1.48 times (95%CI [1.45; 1.50]; crude median cost of stay [IQR] €25,024 [18,898.2 – 35,476.9] for stays with AKI *vs.* €18,034.6 [14,500.5 -23,033.2] for matched stays with no AKI, *p* < 0.0001) and the LOS by 1.47 times (95%CI [1.44; 1.51]; crude median LOS [IQR] 16 [11 – 29] days for stays with AKI *vs.* 11 [9-17] days for matched stays with no AKI, *p* < 0.0001). In the MOV group, the presence of AKI increased the hospital cost by 1.68 times (95%CI [1.65; 1.70]; crude median cost of stay [IQR] €18,587.8 [11,411.9 – 29,546.4] for stays with AKI *vs.* €11,292.6 [7.534,7 -17.661,2] for matched stays with no AKI, *p* < 0.0001) and the LOS by 1.41 times (95%CI [1.38; 1.44]; crude median LOS [IQR] 19 [11 – 36] days for stays with AKI *vs.* 13 [6-23] days for matched stays with no AKI, *p* < 0.0001; Fig. 2).

## Discussion

This cross-sectional nationwide study confirmed the high economic burden for the national health insurance associated with AKI among in-hospitalized patients. In 2018, a diagnostic code for AKI was found in almost half a million hospital stays in France. Compared to patients without an AKI code, those with AKI had a higher consumption of medical procedures including RRT, a higher rate of ICU admissions, and an increased LOS. All of this may have participated in the increase in costs observed.

After adjusting for confounding factors, when AKI was present, an increase in costs of 70%, 68%, and 48% was estimated in patients with sepsis, MOV, and CVEC, respectively. Similarly, AKI increased the LOS by 33%, 41%, and 47% across the three subgroups, respectively.

In the present study, AKI incidence was only 1,5% in the whole population, reaching 2.2% when excluding repeated stays for medical procedures (358,057 AKI stays out of 17,459,379 stays). Although a similar incidence of AKI has been previously reported in studies conducted retrospectively from large databases, it contrasts with the higher incidence of AKI reported when prospective methodologies are used [24]. This suggests an under declaration of AKI by clinicians in the billing charts and highlights the need to increase the efforts towards recognizing and coding AKI more systematically as it largely impacts costs, LOS, and outcomes.

Concerning subpopulations, AKI incidence in surgical stays was similar to the results observed in a recent epidemiological study [14]. For sepsis, we identified a higher number of stays compared with two recent studies using the same discharge database [21, 25]. This can be explained by inclusion of all PMSI stays in our study, unlike previous studies which made a selection; but also by our greater number of explicit codes used to identify sepsis-stays. Furthermore, our population of sepsis patients had a lower risk of AKI, and stays were less expensive compared to previously reported results [13, 25]. This suggests that we may have selected sepsis patients with a lower severity, particularly by including the code “R65.0” in our selection algorithm. This code identifies stays with an infection and a systemic inflammatory response without organ dysfunction. Our results may therefore underestimate the incidence and cost of AKI in septic patients as defined by the latest definition of sepsis, which requires the presence of an organ dysfunction [26].

Among the three observed subgroups, the highest increase in costs associated with AKI was observed in the sepsis group (+ 70%), whereas the most expensive and the longest stays were observed in the CVEC and MOV stays with AKI. Thus, although AKI in surgical patients is less frequent than AKI in patients with sepsis, the former should also be a major concern for policymakers. Bundles of nephroprotective measures have been successfully tested in surgical populations and their use should be promoted in these populations [15, 16].

Herein, a higher incidence of AKI was observed in public hospitals compared to private hospitals and may reflect the greater complexity and severity of the case-mix in public hospital compare to the one in private hospitals.

Previous studies found that up to 45% of AKI patients have not fully recovered their renal function at discharge and are at higher risk of chronic kidney disease, cardiovascular diseases, and death within five years [27, 28]. In addition to initial hospital costs, AKI may therefore be associated with a long-term increase in costs [11]. Although this study was not designed to assess the long-term financial consequences of AKI, it found that patients with AKI were less frequently discharged to home, suggesting that an increase in costs is to be supposed already immediately after the hospital stay.

To the best of our knowledge, the present study is the most recent and largest economic study evaluating the financial burden of AKI in a national insurance health system. A nationwide population was included without further selection, allowing for a pragmatic analysis. In addition to the broad description of costs in the whole population, the case–control analysis provided more precise estimates of the increase in costs associated with AKI, accounting for confounding factors, in different populations. Finally, in 2018, the PMSI was filled with updated ICD-10 codes, using an improved and easier classification of AKI, probably reducing the misclassifications of AKI compared to the previously used ICD-9 codes.

Nevertheless, the present study has certain limitations. First, the severity of AKI and its cause cannot be determined using the PMSI. Because dates cannot be entered along with ICD-10 codes, causal relationship between diseases cannot be assessed from this database. For example, if a stay is associated with an AKI and a sepsis code, it is impossible to know what disease occurred first or if they occurred at the same time. Second, the quality (exhaustivity and exactitude) of the codes entered may be questioned. The correlation between AKI codes in databases used for billing purposes, such as the PMSI, and clinically confirmed AKI is largely debated. In a previous study, Waikar et al*.* found that the ICD-9 codes used for AKI had a low sensitivity of 35.4%, but a very high specificity of 97.7% to diagnose creatinine-confirmed AKI. Therefore, they suggested that the observed prevalence of AKI out of medical claims filled with ICD-9 codes may be underestimated. They also pointed out that false negatives mainly concern small changes in serum creatinine, that might be unnoticed or not perceived as significant by the medical team [29]. Recent studies however, demonstrated that even mild or subclinical AKI might influence the clinical outcomes and should therefore be considered [30]. Third, the AKI-related costs estimated in the present study were only costs, reimbursed by the national health insurance to the hospitals for the stay. Costs were not evaluated from the patient or hospital point of view and the long-term expenses, occurring after the end of the hospital stay, were not investigated. However, additional costs occurring in case of ICU stay or costly procedures and treatments were also collected, to provide the exact total cost of the stay. Fourth, there was no population selection herein; stays for repeated admissions for medical acts such as chronic dialysis or chemotherapy were not excluded from the study and account for 35% of all stays. This might have led to an underestimation of AKI incidence by including in the denominator stays with extremely low risk of AKI, or the same patient repeatedly. Also, when considering the whole PMSI population, these stays may lead to an increase in the difference between AKI and no-AKI stays, as they are mostly no-AKI stays. However, the estimated costs and economic burden related to AKI stays remain unaffected and reflect the real cost supported by the national medical insurance as it exactly depicts what is billed. We also acknowledge limitations associated with our selection of sepsis stays. We followed ATIH guidelines that were in force in 2018 but may not fully reflect the Sepsis-3 definition. Thus, we may have miss stays with a code for infection *plus* a code for organ dysfunction, leading to an underestimation of the number of sepsis stays. Conversely, as we did not verify the presence of organ dysfunction for each sepsis-stay, we may have considered as sepsis certain cases that did not meet sepsis-3 criteria, leading to the selection of a less severe population. However, even if we had specifically sought organ dysfunctions, we would have been unable to determine whether they occurred simultaneously with the infection (defining sepsis) or not. Finally, this study is primarily an economic study, not an epidemiological one, and its aim was not to identify the incidence, determinants, or outcomes of AKI.

## Conclusions

This population-wide study confirmed the major economic burden associated with in-hospital AKI episodes in a developed country and found that AKI was associated with an increase in costs ranging from 48 to 70% in three subgroups of at-risk patients. This should encourage further investigations to reduce the incidence of AKI, particularly among patients with a high risk of AKI such as those with sepsis or undergoing major surgeries. The cost of these strategies should be balanced with the high costs driven by AKI itself.

### Supplementary Information

**Additional file 1.**

## Data Availability

According to French regulations, the authors cannot share the dataset used for the present study which were used under a MR006 authorization from the French data protection authority (*Commission Nationale de l’Informatique et des Libertés*, CNIL) and contains data from the *Programme de médicalisation des systèmes d'information* (PMSI). However, any person or structure, public or private, for-profit or non-profit, can access these data through the HDH (Health Data Hub https://www.health-data-hub.fr/) after an authorization from the CNIL.

## Abbreviations

AKI: Acute kidney injury
CVEC: Cardiovascular surgery with extracorporeal circulation
ENC: *étude nationale des coûts*, National cost evaluation
DRG: Diagnosis related group
ICD-9: International classification of diseases, 9^th^ revision
ICD-10: International classification of diseases, 10^th^ revision
ICU: Intensive care unit
LOS: Length of stay
MVO: Major visceral open surgery
PMSI: *programme de médicalisation des systèmes d’information*, French national hospital discharge database
RRT: Renal replacement therapy
