# Supplementary material for: Economic burden of in-hospital AKI: a one-year analysis of the nationwide French hospital discharge database
Source: BMC Nephrol. 2023 Nov 21;24:343. doi: 10.1186/s12882-023-03396-8 (PMC10664266; doi:10.1186/s12882-023-03396-8)
Supplement: Supplementary file 1 — Additional file 1. [file 12882_2023_3396_MOESM1_ESM.docx]

| Supplementary table 1a. List of codes from the French medical classification of clinical procedures used to identify cardiovascular surgery with extracorporeal circulation (CVEC) and major open visceral surgery (MOV) identification | |
| --- | --- |
|  |  |
| DAAA001 | Agrandissement d'une communication interventriculaire ou du foramen bulboventriculaire, par thoracotomie avec CEC |
| DAAA002 | Plastie d'agrandissement de l'infundibulum pulmonaire avec section de l'anneau pulmonaire, par thoracotomie avec CEC |
| DAAA003 | Plastie d'agrandissement de l'infundibulum pulmonaire, par thoracotomie avec CEC |
| DAFA003 | Résection d'un anévrisme de la paroi ventriculaire du cœur, par thoracotomie avec CEC |
| DAFA006 | Résection d'un bourrelet musculaire infraaortique pour cardiomyopathie obstructive, par thoracotomie avec CEC |
| DAFA007 | Résection musculaire cardiaque intraventriculaire, par thoracotomie avec CEC |
| DAFA008 | Résection ou consolidation d'une zone myocardique infarcie, par thoracotomie avec CEC |
| DAFA009 | Exérèse d'une tumeur du cœur, par thoracotomie avec CEC |
| DAFA010 | Résection d'une sténose congénitale infraorificielle de l'aorte, par thoracotomie avec CEC |
| DAGA001 | Ablation de corps étranger ou exérèse de végétation ou de caillot intracardiaque, par thoracotomie avec CEC |
| DAMA005 | Correction d'une malformation sténosante intraatriale droite ou gauche congénitale, par thoracotomie avec CEC |
| DAMA900 | Cardiomyoplastie de substitution ventriculaire, par thoracotomie avec CEC |
| DASA001 | Fermeture d'une communication interventriculaire avec pose d'un conduit extracardiaque, par thoracotomie avec CEC |
| DASA002 | Fermeture d'une communication atrioventriculaire sans geste sur le septum interventriculaire, par thoracotomie avec CEC |
| DASA003 | Fermeture d'une communication interatriale, par thoracotomie avec CEC |
| DASA004 | Fermeture d'une communication interventriculaire pour discordance atrioventriculaire et transposition ou malposition des gros vaisseaux, par thoracotomie avec CEC |
| DASA006 | Fermeture d'une rupture du septum interventriculaire cardiaque, par thoracotomie avec CEC |
| DASA007 | Fermeture d'une communication interventriculaire avec correction d'une sténose de l'aorte thoracique horizontale et/ou de l'isthme aortique, avec commissurotomie ou valvectomie pulmonaire, par thoracotomie avec CEC |
| DASA008 | Fermeture d'une communication atrioventriculaire avec geste sur le septum interventriculaire, par thoracotomie avec CEC |
| DASA009 | Fermeture d'une communication interventriculaire avec correction d'une sténose de l'aorte thoracique horizontale et/ou de l'isthme aortique, par thoracotomie avec CEC |
| DASA010 | Fermeture de communications interventriculaires multiples, avec résection musculaire cardiaque intraventriculaire, par thoracotomie avec CEC |
| DASA011 | Fermeture de communications interventriculaires multiples, par thoracotomie avec CEC |
| DASA012 | Fermeture d'une communication interventriculaire sans pose d'un conduit extracardiaque, par thoracotomie avec CEC |
| DASA013 | Fermeture d'une communication atrioventriculaire, avec geste sur le septum interventriculaire et correction d'une sténose de la voie pulmonaire, par thoracotomie avec CEC |
| DASA014 | Fermeture d'une communication interventriculaire avec résection musculaire cardiaque intraventriculaire, par thoracotomie avec CEC |
| DBEA001 | Réinsertion d'une prothèse orificielle cardiaque, par thoracotomie avec CEC |
| DBFA001 | Résection d'une fibrose endocardique, par thoracotomie avec CEC |
| DBFA002 | Thrombectomie de prothèse orificielle cardiaque, par thoracotomie avec CEC |
| DBKA001 | Remplacement de la valve aortique par homogreffe, par thoracotomie avec CEC |
| DBKA002 | Remplacement de la valve atrioventriculaire gauche par prothèse en position non anatomique, par thoracotomie avec CEC |
| DBKA003 | Remplacement de la valve aortique par bioprothèse sans armature, par thoracotomie avec CEC |
| DBKA004 | Remplacement de la valve atrioventriculaire droite par prothèse mécanique ou bioprothèse avec armature, par thoracotomie avec CEC |
| DBKA005 | Remplacement de la valve atrioventriculaire gauche par homogreffe, par thoracotomie avec CEC |
| DBKA006 | Remplacement de la valve aortique par prothèse mécanique ou bioprothèse avec armature, par thoracotomie avec CEC |
| DBKA007 | Remplacement de la valve pulmonaire par prothèse mécanique ou bioprothèse avec armature, par thoracotomie avec CEC |
| DBKA008 | Remplacement de la valve atrioventriculaire droite par homogreffe, par thoracotomie avec CEC |
| DBKA009 | Remplacement de la valve aortique et de la valve atrioventriculaire gauche par prothèse mécanique ou par bioprothèse avec armature, par thoracotomie avec CEC |
| DBKA010 | Remplacement de la valve atrioventriculaire gauche par prothèse mécanique ou bioprothèse avec armature, par thoracotomie avec CEC |
| DBKA011 | Remplacement de la valve aortique par prothèse en position non anatomique, par thoracotomie avec CEC |
| DBKA012 | Remplacement de la valve pulmonaire par homogreffe ou bioprothèse sans armature, par thoracotomie avec CEC |
| DBLA001 | Pose d'un tube valvé ou non entre un ventricule et l'artère pulmonaire, par thoracotomie avec CEC |
| DBLA002 | Pose d'un tube valvé entre un ventricule et l'aorte, par thoracotomie avec CEC |
| DBLA003 | Pose d'un tube valvé entre le ventricule gauche et l'aorte [tube apicoaortique], par thoraco-phréno-laparotomie avec CEC |
| DBMA001 | Reconstruction de la voie aortique par élargissement antérodroit de l'anneau avec remplacement de la valve, par thoracotomie avec CEC |
| DBMA002 | Valvoplastie atrioventriculaire gauche, par thoracotomie avec CEC |
| DBMA003 | Annuloplastie atrioventriculaire gauche, par thoracotomie avec CEC |
| DBMA004 | Reconstruction de la voie aortique par transfert de la valve pulmonaire en position aortique avec reconstruction de la voie pulmonaire, par thoracotomie avec CEC |
| DBMA005 | Reconstruction de l'anneau atrioventriculaire gauche avec remplacement de la valve par homogreffe, par thoracotomie avec CEC |
| DBMA006 | Reconstruction de l'anneau aortique avec remplacement de la valve par bioprothèse sans armature, par thoracotomie avec CEC |
| DBMA007 | Reconstruction de l'anneau atrioventriculaire gauche avec valvoplastie, par thoracotomie avec CEC |
| DBMA008 | Annuloplastie atrioventriculaire droite, par thoracotomie avec CEC |
| DBMA009 | Reconstruction de l'anneau aortique avec remplacement de la valve par prothèse mécanique ou bioprothèse avec armature, par thoracotomie avec CEC |
| DBMA010 | Reconstruction de l'anneau aortique avec remplacement de la valve par homogreffe, par thoracotomie avec CEC |
| DBMA011 | Valvoplastie aortique, par thoracotomie avec CEC |
| DBMA012 | Valvoplastie atrioventriculaire droite, par thoracotomie avec CEC |
| DBMA013 | Reconstruction de l'anneau atrioventriculaire gauche avec remplacement de la valve par prothèse mécanique ou bioprothèse avec armature, par thoracotomie avec CEC |
| DBMA015 | Reconstruction de la voie aortique par élargissement antérogauche de l'anneau et ouverture de l'infundibulum pulmonaire, avec remplacement de la valve, par thoracotomie avec CEC |
| DBPA001 | Commissurotomie [Valvulotomie] ou valvectomie [valvulectomie] pulmonaire avec fermeture d'une communication interatriale, par thoracotomie avec CEC |
| DBPA002 | Commissurotomie ou valvectomie atrioventriculaire droite, par thoracotomie avec CEC |
| DBPA004 | Commissurotomie ou valvectomie pulmonaire, par thoracotomie avec CEC |
| DBPA006 | Commissurotomie de la valve atrioventriculaire gauche, par thoracotomie avec CEC |
| DBPA007 | Commissurotomie de la valve aortique, par thoracotomie avec CEC |
| DDAA002 | Angioplastie d'élargissement du tronc commun de l'artère coronaire gauche, par thoracotomie avec CEC |
| DDEA001 | Réimplantation d'une artère coronaire pour anomalie congénitale d'origine, par thoracotomie avec CEC |
| DDMA003 | Revascularisation coronaire par 3 greffons artériels avec 3 anastomoses distales, par thoracotomie avec CEC |
| DDMA004 | Revascularisation coronaire par greffon veineux avec 4 anastomoses distales ou plus, par thoracotomie avec CEC |
| DDMA005 | Revascularisation coronaire par 2 greffons artériels et par greffon veineux avec 3 anastomoses distales, par thoracotomie avec CEC |
| DDMA006 | Revascularisation coronaire par 2 greffons artériels avec 3 anastomoses distales, par thoracotomie avec CEC |
| DDMA007 | Revascularisation coronaire par greffon veineux avec une anastomose distale, par thoracotomie avec CEC |
| DDMA008 | Revascularisation coronaire par 2 greffons artériels avec 4 anastomoses distales ou plus, par thoracotomie avec CEC |
| DDMA009 | Revascularisation coronaire par 2 greffons artériels et par greffon veineux avec 4 anastomoses distales ou plus, par thoracotomie avec CEC |
| DDMA011 | Revascularisation coronaire par un greffon artériel et par greffon veineux avec 2 anastomoses distales, par thoracotomie avec CEC |
| DDMA012 | Revascularisation coronaire par 3 greffons artériels et par greffon veineux avec 4 anastomoses distales ou plus, par thoracotomie avec CEC |
| DDMA013 | Revascularisation coronaire par 3 greffons artériels avec 4 anastomoses distales ou plus, par thoracotomie avec CEC |
| DDMA015 | Revascularisation coronaire par un greffon artériel avec une anastomose distale, par thoracotomie avec CEC |
| DDMA016 | Revascularisation coronaire par greffon veineux avec 3 anastomoses distales, par thoracotomie avec CEC |
| DDMA017 | Revascularisation coronaire par un greffon artériel avec 2 anastomoses distales, par thoracotomie avec CEC |
| DDMA018 | Revascularisation coronaire par un greffon artériel et par greffon veineux avec 3 anastomoses distales, par thoracotomie avec CEC |
| DDMA019 | Revascularisation coronaire par greffon veineux avec 2 anastomoses distales, par thoracotomie avec CEC |
| DDMA020 | Revascularisation coronaire par 2 greffons artériels avec 2 anastomoses distales, par thoracotomie avec CEC |
| DDMA021 | Revascularisation coronaire par un greffon artériel et par greffon veineux avec 4 anastomoses distales ou plus, par thoracotomie avec CEC |
| DDSA001 | Fermeture de fistule coronarocardiaque, par thoracotomie avec CEC |
| DEFA001 | Exérèse ou destruction de foyer arythmogène pour tachycardie ventriculaire, par thoracotomie avec CEC |
| DEFA002 | Exérèse ou destruction de foyer arythmogène ou de faisceau cardionecteur pour tachycardie supraventriculaire, par thoracotomie avec CEC |
| DFAA002 | Angioplastie d'élargissement d'une sténose congénitale d'une branche de l'artère pulmonaire, par thoracotomie avec CEC |
| DFAA003 | Angioplastie d'élargissement d'une sténose congénitale du tronc de l'artère pulmonaire, par thoracotomie avec CEC |
| DFAA004 | Angioplastie d'élargissement d'une sténose congénitale de la bifurcation de l'artère pulmonaire, par thoracotomie avec CEC |
| DFCA004 | Anastomose cavopulmonaire ou atriopulmonaire totale, par thoracotomie avec CEC |
| DFCA006 | Anastomose entre le tronc de l'artère pulmonaire et l'aorte thoracique ascendante, par thoracotomie avec CEC |
| DFCA007 | Anastomose cavopulmonaire bidirectionnelle, par thoracotomie avec CEC |
| DFCA008 | Anastomose cavopulmonaire fonctionnellement terminoterminale, par thoracotomie avec CEC |
| DFCA010 | Anastomose entre le tronc pulmonaire et l'aorte avec anastomose systémicopulmonaire, par thoracotomie avec CEC |
| DFFA001 | Embolectomie de l'artère pulmonaire, par thoracotomie avec CEC |
| DFFA001 | Embolectomie de l'artère pulmonaire, par thoracotomie avec CEC |
| DFFA003 | Thromboendartériectomie du tronc et/ou des branches de l'artère pulmonaire, par thoracotomie avec CEC |
| DFFA003 | Thromboendartériectomie du tronc et/ou des branches de l'artère pulmonaire, par thoracotomie avec CEC |
| DFGA001 | Ablation d'un cerclage pulmonaire, par thoracotomie avec CEC |
| DFGA002 | Ablation d'un cerclage pulmonaire avec fermeture de communications interventriculaires multiples, par thoracotomie avec CEC |
| DFGA004 | Ablation d'un cerclage pulmonaire avec fermeture d'une communication interventriculaire unique, par thoracotomie avec CEC |
| DFMA001 | Réparation d'une obstruction du retour veineux pulmonaire, par thoracotomie avec CEC |
| DFMA004 | Réparation du retour veineux pulmonaire anormal total, par thoracotomie avec CEC |
| DFMA006 | Unifocalisation artérielle pulmonaire, par thoracotomie avec CEC |
| DFMA008 | Réparation du retour veineux pulmonaire anormal partiel, par thoracotomie avec CEC |
| DFMA010 | Démontage d'une anastomose cavopulmonaire totale, par thoracotomie avec CEC |
| DFMA011 | Réparation d'une atrésie de l'artère pulmonaire avec fermeture d'une communication interventriculaire avec prothèse [tube valvé ou non], par thoracotomie avec CEC |
| DFMA012 | Réparation d'une atrésie de l'artère pulmonaire avec fermeture d'une communication interventriculaire sans prothèse [tube valvé ou non], par thoracotomie avec CEC |
| DGAA002 | Angioplastie d'élargissement ou résection-anastomose d'une sténose de l'aorte thoracique horizontale et de l'isthme aortique avec réparation de lésion intracardiaque, par thoracotomie avec CEC |
| DGAA003 | Angioplastie d'élargissement de l'aorte thoracique horizontale avec réimplantation des troncs supraaortiques, par thoracotomie avec CEC |
| DGAA004 | Angioplastie d'élargissement ou résection-anastomose d'une sténose de l'aorte thoracique horizontale et de l'isthme aortique, par thoracotomie avec CEC |
| DGAA006 | Angioplastie d'élargissement de l'aorte thoracique horizontale sans réimplantation des troncs supraaortiques, par thoracotomie avec CEC |
| DGCA006 | Suture d'une rupture de l'isthme de l'aorte, par thoracotomie avec CEC |
| DGCA011 | Pontages multiples entre l'aorte et les troncs supraaortiques, par thoracotomie avec CEC |
| DGCA017 | Suture latérale de plaie de l'aorte thoracique, par thoracotomie avec CEC |
| DGCA027 | Pontage entre l'aorte thoracique ascendante et l'aorte abdominale sans exclusion de l'aorte thoracique descendante, par thoracotomie et par laparotomie avec CEC |
| DGCA028 | Pontage entre l'aorte thoracique ascendante et l'aorte thoracique descendante, par thoracotomie avec CEC |
| DGFA002 | Thromboendartériectomie de l'aorte thoracique horizontale et des troncs supraaortiques, par thoracotomie avec CEC |
| DGFA017 | Résection-anastomose de l'aorte thoracique descendante ou de l'aorte juxtadiaphragmatique, par thoracotomie avec CEC |
| DGFA018 | Résection-anastomose de l'isthme de l'aorte, par thoracotomie avec CEC |
| DGKA001 | Remplacement de l'aorte thoracique ascendante et de l'aorte horizontale sans remplacement de la valve aortique, sans réimplantation des artères coronaires, par thoracotomie avec CEC |
| DGKA003 | Remplacement de l'aorte thoracique ascendante sans remplacement de la valve aortique, avec réimplantation des artères coronaires, par thoracotomie avec CEC |
| DGKA005 | Remplacement de l'aorte thoracique horizontale, par thoracotomie avec CEC |
| DGKA007 | Remplacement de l'aorte thoracique descendante, par thoracotomie avec CEC |
| DGKA008 | Remplacement de l'aorte thoracique descendante et de l'aorte juxtadiaphragmatique, par thoraco-phréno-laparotomie avec CEC |
| DGKA010 | Remplacement de l'aorte juxtadiaphragmatique, par thoraco-phréno-laparotomie avec CEC |
| DGKA011 | Remplacement de l'aorte thoracique ascendante avec remplacement de la valve aortique, sans réimplantation des artères coronaires, par thoracotomie avec CEC |
| DGKA012 | Remplacement de l'ensemble de l'aorte thoracique, par thoraco-phréno-laparotomie avec CEC |
| DGKA014 | Remplacement de l'aorte thoracique ascendante et de l'aorte horizontale avec remplacement de la valve aortique, avec réimplantation des artères coronaires, par thoracotomie avec CEC |
| DGKA015 | Remplacement de l'aorte thoracique ascendante avec remplacement de la valve aortique, avec réimplantation des artères coronaires, par thoracotomie avec CEC |
| DGKA018 | Remplacement de l'aorte thoracique ascendante et de l'aorte horizontale avec remplacement de la valve aortique, sans réimplantation des artères coronaires, par thoracotomie avec CEC |
| DGKA021 | Remplacement de l'aorte thoracique descendante pour sténose congénitale, par thoracotomie avec CEC |
| DGKA023 | Remplacement de l'isthme de l'aorte, par thoracotomie avec CEC |
| DGKA024 | Remplacement de l'isthme de l'aorte pour coarctation, par thoracotomie avec CEC |
| DGKA025 | Remplacement de l'aorte thoracique ascendante sans remplacement de la valve aortique, sans réimplantation des artères coronaires, par thoracotomie avec CEC |
| DGKA026 | Remplacement de l'aorte thoracique ascendante et de l'aorte horizontale sans remplacement de la valve aortique, avec réimplantation des artères coronaires, par thoracotomie avec CEC |
| DGKA027 | Remplacement de l'aorte thoracique descendante pour rupture d'anévrisme, par thoracotomie avec CEC |
| DGKA028 | Remplacement de l'aorte thoracique ascendante pour rupture d'anévrisme, par thoracotomie avec CEC |
| DGKA029 | Remplacement de l'aorte juxtadiaphragmatique pour rupture d'anévrisme, par thoraco-phréno-laparotomie avec CEC |
| DGMA002 | Correction d'une interruption de l'aorte thoracique horizontale sans prothèse, avec réparation du tronc artériel commun [truncus arteriosus], par thoracotomie avec CEC |
| DGMA005 | Correction d'une interruption de l'aorte thoracique horizontale avec réparation de lésion intracardiaque associée, par thoracotomie avec CEC |
| DGMA009 | Plastie d'une sténose supraorificielle de l'aorte, par thoracotomie avec CEC |
| DGMA010 | Correction d'une interruption de l'aorte thoracique horizontale avec prothèse, par thoracotomie avec CEC |
| DGMA012 | Correction d'une interruption de l'aorte thoracique horizontale sans prothèse, par thoracotomie avec CEC |
| DGSA003 | Fermeture d'une fenêtre [fistule] aortopulmonaire, par thoracotomie avec CEC |
| DZEA001 | Transplantation itérative du cœur, par thoracotomie avec CEC |
| DZEA002 | Transplantation orthotopique du cœur, par thoracotomie avec CEC |
| DZEA003 | Transplantation hétérotopique du cœur, par thoracotomie avec CEC |
| DZEA004 | Transplantation du bloc cœur-poumons, par thoracotomie avec CEC |
| DZFA004 | Exérèse d'un greffon de transplantation du cœur avec pose d'une assistance circulatoire mécanique biventriculaire interne, par thoracotomie avec CEC |
| DZMA001 | Réparation d'une malposition des gros vaisseaux avec communication interventriculaire, sans conduit extracardiaque ni mobilisation de l'artère pulmonaire, par thoracotomie avec CEC |
| DZMA002 | Réparation anatomique de la discordance atrioventriculaire et de la transposition ou malposition des gros vaisseaux avec communication interventriculaire et sténose pulmonaire à l'étage ventriculaire et atrial, par thoracotomie avec CEC |
| DZMA003 | Réparation anatomique à l'étage artériel et atrial de la discordance atrioventriculaire et de la transposition ou malposition des gros vaisseaux avec communication interventriculaire, par thoracotomie avec CEC |
| DZMA004 | Réparation à l'étage artériel d'une transposition ou d'une malposition des gros vaisseaux avec fermeture d'une communication interventriculaire, par thoracotomie avec CEC |
| DZMA005 | Réparation de la tétralogie de Fallot sans section de l'anneau pulmonaire, par thoracotomie avec CEC |
| DZMA006 | Réparation à l'étage atrial d'une transposition ou d'une malposition des gros vaisseaux avec fermeture d'une communication interventriculaire, par thoracotomie avec CEC |
| DZMA007 | Réparation à l'étage artériel de la transposition des gros vaisseaux, par thoracotomie avec CEC |
| DZMA009 | Réparation du tronc artériel commun [truncus arteriosus], par thoracotomie avec CEC |
| DZMA010 | Réparation d'une malposition des gros vaisseaux avec communication interventriculaire, par pose de conduit extracardiaque ou mobilisation de l'artère pulmonaire, par thoracotomie avec CEC |
| DZMA011 | Réparation de la tétralogie de Fallot avec section de l'anneau pulmonaire, par thoracotomie avec CEC |
| DZMA012 | Réparation à l'étage atrial de la transposition des gros vaisseaux, par thoracotomie avec CEC |
| DZSA001 | Suppression d'une anastomose palliative pour cardiopathie congénitale, avec angioplastie d'agrandissement d'une branche de l'artère pulmonaire, par thoracotomie avec CEC |
| EPMA001 | Correction d'une anomalie du retour veineux systémique sans prothèse, par thoracotomie avec CEC |
| EPMA002 | Correction d'une anomalie du retour veineux systémique avec prothèse, par thoracotomie avec CEC |
| EQGA004 | Ablation d'un dispositif d'assistance circulatoire interne ou externe, en dehors d'un dispositif de contrepulsion diastolique intraaortique, par thoracotomie avec CEC |
| EQKA002 | Changement d'un système d'assistance circulatoire mécanique ventriculaire, par thoracotomie avec CEC |
| EQLA004 | Pose d'une assistance circulatoire mécanique monoventriculaire externe, par thoracotomie avec CEC |
| EQLA006 | Pose d'une assistance circulatoire mécanique biventriculaire externe, par thoracotomie avec CEC |
| EQLA008 | Pose d'une assistance circulatoire mécanique monoventriculaire interne, par thoracotomie avec CEC |
| EQLA010 | Pose d'une prothèse mécanique biventriculaire orthotopique, par thoracotomie avec CEC |
| GEFA013 | Résection-anastomose de la trachée pour sténose congénitale de la trachée, par thoracotomie avec CEC |
| GEFA013 | Résection-anastomose de la trachée pour sténose congénitale de la trachée, par thoracotomie avec CEC |
| GFEA001 | Transplantation séquentielle des 2 poumons, par thoracotomie avec CEC |
| GFEA002 | Transplantation de lobe pulmonaire, par thoracotomie avec CEC |
| GFEA006 | Transplantation bipulmonaire, par thoracotomie avec CEC |
| GFEA007 | Transplantation d'un poumon, par thoracotomie avec CEC |
|  |  |
| ADPA002 | Vagotomie tronculaire postérieure avec séromyotomie œsogastrique antérieure, par laparotomie |
| ADPA014 | Vagotomie tronculaire, par thoracotomie |
| ADPA018 | Vagotomie tronculaire avec gastrojéjunostomie, par laparotomie |
| ADPA019 | Vagotomie suprasélective, par laparotomie |
| ADPA022 | Vagotomie tronculaire, par laparotomie |
| ADPA024 | Vagotomie tronculaire avec antrectomie gastrique, par laparotomie |
| AJNA001 | Destruction chimique unilatérale ou bilatérale de la chaîne splanchnique, par laparotomie |
| EDPA002 | Libération d'une artère digestive, par laparotomie |
| EDSA001 | Ligature d'une artère digestive, par laparotomie |
| EDSA003 | Ligature des artères iliaques internes, par laparotomie |
| FCFA006 | Curage lymphonodal [ganglionnaire] pelvien, par laparotomie |
| FCFA006 | Curage lymphonodal [ganglionnaire] pelvien, par laparotomie |
| FCFA010 | Curage lymphonodal [ganglionnaire] lomboaortique, par laparotomie |
| FCFA010 | Curage lymphonodal [ganglionnaire] lomboaortique, par laparotomie |
| FCFA019 | Curage lymphonodal [ganglionnaire] iliaque unilatéral ou bilatéral, par laparotomie |
| FCFA019 | Curage lymphonodal [ganglionnaire] iliaque unilatéral ou bilatéral, par laparotomie |
| FCFA022 | Curage lymphonodal [ganglionnaire] lomboaortique avec curage iliaque unilatéral ou bilatéral, par laparotomie |
| FCFA022 | Curage lymphonodal [ganglionnaire] lomboaortique avec curage iliaque unilatéral ou bilatéral, par laparotomie |
| FFFA001 | Splénectomie totale, par laparotomie |
| HASA012 | Fermeture d'orostome ou de pharyngostome |
| HDFA012 | Pharyngectomie postérieure, par cervicotomie |
| HDFA014 | Résection de récidive de diverticule pharyngoœsophagien, par cervicotomie |
| HDFA015 | Résection de diverticule pharyngoœsophagien avec myotomie extramuqueuse, par cervicotomie |
| HDFA016 | Pharyngectomie latérale, par cervicotomie |
| HDGA001 | Ablation de corps étranger pharyngoœsophagien, par cervicotomie |
| HECA001 | Suture de plaie ou de perforation de l'œsophage, par cervicotomie |
| HECA002 | Suture de plaie ou de perforation de l'œsophage, par thoracotomie |
| HECA003 | Exclusion bipolaire de l'œsophage, par cervicotomie et par laparotomie |
| HECA004 | Suture de plaie ou de perforation de l'œsophage, par laparotomie |
| HECA005 | Œsophagostomie cutanée, par cervicotomie |
| HEDA001 | Diverticulopexie de l'œsophage, par cervicotomie |
| HEFA001 | Œsophagectomie avec œsophagogastroplastie, par thoracophrénotomie gauche |
| HEFA002 | Œsophagectomie avec œsophagogastroplastie, par cervicotomie, thoracotomie et laparotomie |
| HEFA003 | Œsophagectomie avec œsophagogastroplastie, par thoracotomie et par cœlioscopie |
| HEFA004 | Œsophagectomie totale avec œsophagogastroplastie, par cervicotomie et par laparotomie |
| HEFA005 | Œsophagectomie avec œsophagojéjunostomie, par thoraco-phréno-laparotomie |
| HEFA006 | Œsophagectomie totale avec œsophagocoloplastie, par cervicotomie et par laparotomie |
| HEFA007 | Œsophagectomie avec œsophagocoloplastie, par cervicotomie, thoracotomie et laparotomie |
| HEFA008 | Œsophago-pharyngo-laryngectomie totale avec œsophagogastroplastie, par cervicotomie et par laparotomie |
| HEFA009 | Œsophagectomie avec œsophagocoloplastie, par thoracotomie et par laparotomie |
| HEFA010 | Exérèse de duplication de l'œsophage, par thoracotomie et par laparotomie |
| HEFA011 | Œsophagectomie avec œsophagojéjunostomie, par thoracotomie et par laparotomie |
| HEFA012 | Œsophagectomie avec œsophagogastroplastie, par thoracotomie et par laparotomie |
| HEFA013 | Œsophagectomie avec œsophagogastroplastie, par thoraco-phréno-laparotomie |
| HEFA014 | Exérèse de duplication de l'œsophage, par thoracotomie |
| HEFA015 | Résection de diverticule de l'œsophage, par thoracotomie |
| HEFA016 | Œsophagectomie avec œsophagocoloplastie, par thoraco-phréno-laparotomie |
| HEFA017 | Œsophago-pharyngo-laryngectomie totale avec œsophagocoloplastie, par cervicotomie et par laparotomie |
| HEFA018 | Œsophagectomie avec œsophagogastroplastie, par cervicotomie, thoracotomie et cœlioscopie |
| HEFA019 | Excision de tumeur de l'œsophage sans interruption de la continuité, par thoracotomie |
| HEFA020 | Œsophagectomie totale sans rétablissement de la continuité, par cervicotomie et par laparotomie |
| HEFA021 | Résection-anastomose de rétrécissement congénital de l'œsophage, par thoracotomie |
| HEFA022 | Œsophagectomie totale sans rétablissement de la continuité, par thoracotomie |
| HEFC800 | Oesophagectomie avec oesophagogastroplastie, par thoracoscopie et laparotomie ou coelioscopie |
| HEFC801 | Oesophagectomie avec oesophagogastroplastie, par cervicotomie, thoracoscopie et laparotomie ou coelioscopie |
| HEMA001 | Œsophagocoloplastie rétrosternale sans œsophagectomie, avec anastomose œsophagocolique cervicale, par cervicotomie et par laparotomie |
| HEMA002 | Reconstruction secondaire de l'œsophage pour atrésie avec fistule, par thoracotomie |
| HEMA003 | Reconstruction de l'œsophage et fermeture de la fistule en un temps pour atrésie avec fistule, par thoracotomie |
| HEMA004 | Reconstruction de l'œsophage en un temps pour atrésie sans fistule, par thoracotomie |
| HEMA005 | Œsophagocoloplastie rétrosternale sans œsophagectomie, avec anastomose pharyngocolique, par cervicotomie et par laparotomie |
| HEMA006 | Reconstruction de l'œsophage par lambeau libre de segment digestif, avec anastomoses vasculaires et digestives |
| HEMA007 | Plastie de sténose anastomotique de l'œsophage, par cervicotomie |
| HEMA008 | Œsophagogastroplastie rétrosternale sans œsophagectomie, avec anastomose œsophagogastrique cervicale, par cervicotomie et par laparotomie |
| HEMA009 | Œsophagogastroplastie rétrosternale sans œsophagectomie, avec anastomose pharyngogastrique, par cervicotomie et par laparotomie |
| HEPA001 | Œso-cardio-myotomie extramuqueuse sans réalisation de procédé antireflux, par thoracotomie |
| HEPA002 | Œso-cardio-myotomie extramuqueuse avec réalisation de procédé antireflux, par laparotomie |
| HEPA003 | Œso-cardio-myotomie extramuqueuse avec réalisation de procédé antireflux, par thoracotomie |
| HEPA004 | Transsection de l'œsophage avec splénectomie et déconnexion portosystémique, par laparotomie |
| HEPA005 | Transsection de l'œsophage, par thoracotomie ou par laparotomie |
| HEPA006 | Œso-cardio-myotomie extramuqueuse sans réalisation de procédé antireflux, par laparotomie |
| HEPA007 | Transsection de l'œsophage avec splénectomie et déconnexion portosystémique, par thoracotomie et par laparotomie |
| HESA001 | Fermeture de fistule œsotrachéale acquise, par cervicotomie |
| HESA002 | Fermeture d'une fistule œsotrachéale congénitale sans atrésie de l'œsophage, par cervicotomie et par thoracotomie |
| HESA004 | Fermeture de fistule œsotrachéale acquise, par cervicothoracotomie ou par thoracotomie |
| HESA005 | Fermeture de fistule cutanée de l'œsophage, par cervicotomie |
| HESA006 | Fermeture d'une fistule œsotrachéale congénitale sans atrésie de l'œsophage, par cervicotomie |
| HESA007 | Fermeture de fistule œsotrachéale acquise avec résection de la trachée, par cervicothoracotomie ou par thoracotomie |
| HESA008 | Fermeture de fistule œsotrachéale acquise avec résection-anastomose de la trachée, par cervicotomie |
| HESA009 | Fermeture de la fistule œsotrachéale d'une atrésie de l'œsophage, par thoracotomie |
| HFCA001 | Court-circuit [Bypass] gastrique pour obésité morbide, par laparotomie |
| HFCA002 | Gastrostomie cutanée, par laparotomie |
| HFCA003 | Suture de plaie ou de perforation de l'estomac ou du duodénum, par laparotomie |
| HFCA004 | Gastrojéjunostomie de dérivation [Gastro-entéro-anastomose sans résection gastrique], par laparotomie |
| HFDA001 | Gastropexie chez l'enfant, par laparotomie |
| HFDA002 | Gastropexie postérieure [Cardiopexie], par laparotomie |
| HFFA001 | Gastrectomie avec court-circuit biliopancréatique ou intestinal pour obésité morbide, par laparotomie |
| HFFA002 | Gastrectomie partielle inférieure avec anastomose gastroduodénale, par laparotomie |
| HFFA003 | Gastrectomie partielle supérieure [polaire supérieure] avec rétablissement de la continuité, par laparotomie |
| HFFA004 | Résection de duplication gastroduodénale avec dérivation du conduit biliaire commun, par laparotomie |
| HFFA005 | Gastrectomie totale avec rétablissement de la continuité, par laparotomie |
| HFFA006 | Gastrectomie partielle inférieure avec anastomose gastrojéjunale, par laparotomie |
| HFFA007 | Résection de duplication gastroduodénale sans dérivation du conduit biliaire commun, par laparotomie |
| HFFA008 | Dégastrogastrectomie partielle avec rétablissement de la continuité, par laparotomie |
| HFFA009 | Résection partielle atypique de la paroi de l'estomac n'interrompant pas la continuité, par laparotomie |
| HFFA010 | Résection de duplication gastrique, par laparotomie |
| HFFA011 | Gastrectomie longitudinale [Sleeve gastrectomy] pour obésité morbide, par laparotomie |
| HFKA002 | Changement d'un anneau ajustable périgastrique pour obésité morbide, par laparotomie |
| HFMA001 | Confection d'une valve tubérositaire sans libération de la grande courbure gastrique, par thoracotomie |
| HFMA003 | Confection d'une valve tubérositaire sans libération de la grande courbure gastrique, par laparotomie |
| HFMA004 | Pyloroplastie ou duodénoplastie, par laparotomie |
| HFMA005 | Totalisation secondaire de gastrectomie avec rétablissement de la continuité, par laparotomie |
| HFMA007 | Confection d'une valve tubérositaire avec libération de la grande courbure gastrique, par thoracotomie |
| HFMA008 | Confection d'une valve tubérositaire avec libération de la grande courbure gastrique, par laparotomie |
| HFMA009 | Gastroplastie par pose d'anneau ajustable périgastrique pour obésité morbide, par laparotomie |
| HFMA010 | Gastroplastie verticale calibrée pour obésité morbide, par laparotomie |
| HFMA011 | Repositionnement ou ablation d'un anneau ajustable périgastrique, par laparotomie |
| HFPA001 | Gastrotomie à visée thérapeutique, par laparotomie |
| HFPA002 | Pylorotomie extramuqueuse [Pyloromyotomie extramuqueuse], par laparotomie |
| HFPA003 | Gastrotomie exploratrice, par laparotomie |
| HGAA002 | Plastie d'allongement de l'intestin grêle, par laparotomie |
| HGAA003 | Plastie d'élargissement unique ou multiple de l'intestin grêle, par laparotomie |
| HGCA001 | Entéroentérostomie de dérivation, par laparotomie |
| HGCA002 | Suture de plaie ou de perforation de l'intestin grêle, par laparotomie |
| HGCA003 | Anastomose duodénoduodénale pour atrésie ou sténose congénitale du duodénum, par laparotomie |
| HGCA005 | Iléocolostomie de dérivation [Anastomose iléocolique sans exérèse intestinale], par laparotomie |
| HGCA006 | Dérivation des sécrétions duodénales et biliopancréatiques par réfection du montage après gastrectomie [Diversion du duodénum], par laparotomie |
| HGCA007 | Exclusion du duodénum, par laparotomie |
| HGCA008 | Entérostomie cutanée, par laparotomie |
| HGCA009 | Court-circuit biliopancréatique ou intestinal pour obésité morbide, par laparotomie |
| HGEA001 | Détorsion intestinale ou section de bride péritonéale pour vice de rotation de l'anse intestinale primitive [mesenterium commune], par laparotomie |
| HGEA002 | Transplantation d'intestin grêle et de foie réduit, par laparotomie |
| HGEA004 | Transplantation d'intestin grêle et de foie total, par laparotomie |
| HGEA005 | Transplantation d'intestin grêle, par laparotomie |
| HGFA001 | Résection de l'angle duodénojéjunal avec rétablissement de la continuité, par laparotomie |
| HGFA003 | Résection segmentaire unique de l'intestin grêle sans rétablissement de la continuité, en dehors de l'occlusion, par laparotomie |
| HGFA004 | Résection segmentaire multiple de l'intestin grêle, par laparotomie |
| HGFA005 | Résection segmentaire unique de l'intestin grêle pour occlusion, par laparotomie |
| HGFA007 | Résection segmentaire unique de l'intestin grêle avec rétablissement de la continuité, en dehors de l'occlusion, par laparotomie |
| HGFA008 | Résection segmentaire d'une atrésie unique de l'intestin grêle avec rétablissement de la continuité, par laparotomie |
| HGFA009 | Résection segmentaire d'une atrésie étagée de l'intestin grêle avec rétablissement de la continuité, par laparotomie |
| HGFA010 | Exérèse de duplication de l'intestin grêle sans résection intestinale, par laparotomie |
| HGFA011 | Exérèse de duplication de l'intestin grêle avec résection intestinale, par laparotomie |
| HGFA012 | Résection de l'intestin grêle et/ou du côlon pour péritonite néonatale, par laparotomie |
| HGFA013 | Résection totale de l'intestin grêle, par laparotomie |
| HGMA002 | Remise en circuit secondaire du duodénum, par laparotomie |
| HGPA001 | Duodénotomie à visée thérapeutique ou duodénectomie partielle, par laparotomie |
| HGPA002 | Entérotomie à visée thérapeutique, par laparotomie |
| HGPA004 | Libération étendue de l'intestin grêle [Entérolyse étendue] pour occlusion aigüe, par laparotomie |
| HGPA005 | Duodénotomie exploratrice, par laparotomie |
| HGPA006 | Désobstruction de l'intestin grêle pour iléus méconial, par laparotomie |
| HHCA001 | Suture de plaie ou de perforation du côlon, par laparotomie |
| HHCA002 | Colostomie cutanée, par laparotomie |
| HHCA003 | Colocolostomie de dérivation [Anastomose colocolique sans exérèse colique], par laparotomie |
| HHFA002 | Colectomie gauche avec libération de l'angle colique gauche, avec rétablissement de la continuité, par cœlioscopie ou par laparotomie avec préparation par cœlioscopie |
| HHFA003 | Résection du côlon pour malformation congénitale avec rétablissement de la continuité, par laparotomie |
| HHFA004 | Colectomie totale avec conservation du rectum, avec anastomose iléorectale, par cœlioscopie ou par laparotomie avec préparation par cœlioscopie |
| HHFA005 | Colectomie totale avec conservation du rectum, sans rétablissement de la continuité, par cœlioscopie ou par laparotomie avec préparation par cœlioscopie |
| HHFA006 | Colectomie gauche avec libération de l'angle colique gauche, avec rétablissement de la continuité, par laparotomie |
| HHFA008 | Colectomie droite avec rétablissement de la continuité, par cœlioscopie ou par laparotomie avec préparation par cœlioscopie |
| HHFA009 | Colectomie droite avec rétablissement de la continuité, par laparotomie |
| HHFA010 | Colectomie gauche sans libération de l'angle colique gauche, avec rétablissement de la continuité, par cœlioscopie ou par laparotomie avec préparation par cœlioscopie |
| HHFA014 | Colectomie gauche sans libération de l'angle colique gauche, sans rétablissement de la continuité, par laparotomie |
| HHFA017 | Colectomie gauche sans libération de l'angle colique gauche, avec rétablissement de la continuité, par laparotomie |
| HHFA018 | Colectomie transverse, par laparotomie |
| HHFA021 | Colectomie totale avec conservation du rectum, sans rétablissement de la continuité, par laparotomie |
| HHFA022 | Colectomie totale avec conservation du rectum, avec anastomose iléorectale, par laparotomie |
| HHFA023 | Colectomie transverse, par cœlioscopie ou par laparotomie avec préparation par cœlioscopie |
| HHFA024 | Colectomie gauche avec libération de l'angle colique gauche, sans rétablissement de la continuité, par laparotomie |
| HHFA026 | Colectomie droite sans rétablissement de la continuité, par laparotomie |
| HHFA027 | Résection complète d'un prolapsus colorectal extériorisé, avec anastomose coloanale et myorraphie du plancher pelvien |
| HHFA028 | Coloproctectomie totale avec anastomose iléoanale, par cœlioscopie ou par laparotomie avec préparation par cœlioscopie |
| HHFA029 | Coloproctectomie totale sans rétablissement de la continuité, par cœlioscopie ou par laparotomie avec préparation par cœlioscopie |
| HHFA030 | Coloproctectomie totale sans rétablissement de la continuité, par laparotomie |
| HHFA031 | Coloproctectomie totale avec anastomose iléoanale, par laparotomie |
| HHMA002 | Réfection de stomie cutanée intestinale, par laparotomie |
| HHMA003 | Rétablissement secondaire de la continuité digestive après colectomie, par laparotomie |
| HHPA001 | Colotomie à visée thérapeutique, par laparotomie |
| HHSA001 | Fermeture de colostomie cutanée latérale, par abord direct |
| HJBA001 | Plicature de la paroi antérieure du rectum, par abord périnéal |
| HJCA001 | Suture de plaie ou de perforation intrapéritonéale du rectum, par laparotomie |
| HJDA001 | Rectopexie, par laparotomie |
| HJEA001 | Abaissement du rectum avec cervicocystoplastie, urétroplastie et vaginoplastie pour malformation anorectale haute, par laparotomie et par abord périnéal |
| HJEA002 | Abaissement du rectum pour malformation anorectale haute ou intermédiaire, par abord transsacrococcygien [de Kraske] et par abord périnéal |
| HJEA003 | Abaissement du rectum pour malformation anorectale haute, par laparotomie et par abord périnéal |
| HJEA004 | Abaissement du rectum pour malformation anorectale haute ou intermédiaire, par abord périnéal médian |
| HJFA001 | Résection rectocolique avec abaissement colique rétrorectal par laparotomie, avec anastomose colorectale par voie anale |
| HJFA002 | Résection rectosigmoïdienne avec anastomose colorectale infrapéritonéale, par laparotomie |
| HJFA003 | Exérèse de tumeur du rectum, par abord transsphinctérien |
| HJFA004 | Résection rectosigmoïdienne avec anastomose colorectale infrapéritonéale, par cœlioscopie ou par laparotomie avec préparation par cœlioscopie |
| HJFA005 | Amputation du rectum, par abord périnéal |
| HJFA006 | Résection rectosigmoïdienne par laparotomie, avec anastomose coloanale par voie anale ou par abord transsphinctérien |
| HJFA007 | Amputation du rectum, par laparotomie et par abord périnéal |
| HJFA008 | Résection circonférentielle de la muqueuse d'un prolapsus rectal et plicature de la musculeuse, par abord périnéal |
| HJFA009 | Résection circonférentielle de la muqueuse d'un prolapsus rectal et plicature de la musculeuse, avec réduction d'hédrocèle par abord périnéal |
| HJFA010 | Exérèse de duplication du rectum, par abord transsacrococcygien [de Kraske] |
| HJFA011 | Résection rectosigmoïdienne dépassant le cul-de-sac de Douglas, sans rétablissement de la continuité, par laparotomie |
| HJFA012 | Proctectomie secondaire par laparotomie avec anastomose iléoanale par voie transanale, après colectomie totale initiale |
| HJFA013 | Résection d'une fistule rectovaginale acquise, avec fermeture en un temps par suture musculaire et lambeau d'avancement, par abord périnéal |
| HJFA014 | Exérèse de moignon rectal résiduel, par abord périnéal |
| HJFA015 | Résection rectocolique totale pour aganglionose congénitale par laparotomie, avec rétablissement de la continuité par laparotomie ou par voie anale |
| HJFA016 | Résection rectocolique subtotale pour aganglionose congénitale par laparotomie, avec rétablissement de la continuité par laparotomie ou par voie anale |
| HJFA017 | Résection rectosigmoïdienne par cœlioscopie ou par laparotomie avec préparation par cœlioscopie, avec anastomose coloanale par voie anale |
| HJFA018 | Exérèse de tumeur du rectum, par abord transsacrococcygien [de Kraske] |
| HJFA019 | Amputation du rectum, par cœlioscopie ou par laparotomie avec préparation par cœlioscopie et par abord périnéal |
| HJFA020 | Résection rectosigmoïdienne pour aganglionose congénitale par laparotomie, avec rétablissement de la continuité par voie anale |
| HJMA002 | Reconstruction de la paroi antérieure de l'anus et du rectum et de la paroi postérieure du vagin, avec sphinctéroplastie anale, par abord périnéal |
| HJPA001 | Mise à plat d'une fistule rectovaginale acquise, par périnéotomie |
| HJSA001 | Fermeture d'une fistule rectovaginale haute ou colovaginale acquise, par laparotomie |
| HLCA001 | Hépato-porto-cholécystostomie ou hépato-porto-entérostomie pour atrésie des conduits biliaires extrahépatiques, par laparotomie |
| HLCA002 | Fenestration de kystes biliaires hépatiques, par laparotomie |
| HLEA001 | Transplantation de foie total |
| HLEA002 | Transplantation de foie réduit |
| HLFA002 | Résection du dôme saillant de kyste hydatique du foie, par laparotomie |
| HLFA003 | Résection du lobe caudé [de Spigel] [segment I] du foie, par laparotomie |
| HLFA004 | Hépatectomie droite élargie au lobe caudé [de Spigel] [segment I], par laparotomie |
| HLFA005 | Lobectomie hépatique droite [Hépatectomie droite élargie au segment IV], par laparotomie |
| HLFA006 | Trisegmentectomie hépatique, par laparotomie |
| HLFA007 | Hépatectomie gauche élargie au lobe caudé [de Spigel] [segment I], par laparotomie |
| HLFA009 | Bisegmentectomie hépatique, par laparotomie |
| HLFA010 | Hépatectomie centrale, par laparotomie |
| HLFA011 | Lobectomie hépatique gauche, par laparotomie |
| HLFA012 | Kystectomie ou périkystectomie hépatique, par laparotomie |
| HLFA014 | Séquestrectomie hépatique, par laparotomie |
| HLFA015 | Prélèvement d'un greffon hépatique, chez un sujet vivant |
| HLFA017 | Hépatectomie droite, par laparotomie |
| HLFA018 | Hépatectomie gauche, par laparotomie |
| HLFA019 | Résection atypique du foie, par laparotomie |
| HLFA020 | Unisegmentectomie hépatique, par laparotomie |
| HLJA001 | Évacuation de collection hépatique, par laparotomie |
| HLNA007 | Destruction de tumeur hépatique avec courant de radiofréquence, par laparotomie |
| HLSA001 | Hémostase de lésion du foie, par laparotomie |
| HMCA001 | Cholécystoduodénostomie pour atrésie des conduits biliaires extrahépatiques, par laparotomie |
| HMCA002 | Cholédochoduodénostomie, par laparotomie |
| HMCA003 | Cholécystogastrostomie ou cholécystoduodénostomie, par laparotomie |
| HMCA004 | Cholédochoduodénostomie avec gastrojéjunostomie, par laparotomie |
| HMCA005 | Anastomose biliodigestive intrahépatique portant sur un conduit biliaire segmentaire, par laparotomie |
| HMCA006 | Cholédochojéjunostomie, par laparotomie |
| HMCA007 | Anastomose biliodigestive au-dessus de la convergence portant sur plusieurs conduits biliaires, par laparotomie |
| HMCA008 | Anastomose biliodigestive portant sur la convergence des conduits hépatiques, par laparotomie |
| HMCA009 | Cholécystostomie cutanée, par laparotomie |
| HMCA010 | Cholécystogastrostomie ou cholécystoduodénostomie avec gastrojéjunostomie, par laparotomie |
| HMCA011 | Cholécystojéjunostomie, par laparotomie |
| HMFA001 | Cholécystectomie avec cholédochojéjunostomie, par laparotomie |
| HMFA002 | Cholécystectomie avec cholédochogastrostomie ou cholédochoduodénostomie, par laparotomie |
| HMFA003 | Cholécystectomie par cœlioscopie, avec ablation de calcul de la voie biliaire principale par cholédochotomie, par laparotomie |
| HMFA004 | Cholécystectomie avec ablation transcystique de calcul de la voie biliaire principale, par laparotomie |
| HMFA005 | Cholécystectomie par cœlioscopie, avec cholédochojéjunostomie par laparotomie |
| HMFA006 | Cholécystectomie par cœlioscopie, avec cholédochoduodénostomie par laparotomie |
| HMFA007 | Cholécystectomie, par laparotomie |
| HMFA008 | Cholécystectomie avec ablation de calcul de la voie biliaire principale par cholédochotomie, par laparotomie |
| HMFA009 | Résection de la voie biliaire principale pédiculaire avec anastomose biliodigestive, par laparotomie |
| HMFA010 | Résection de la voie biliaire principale pédiculaire et intrapancréatique avec anastomose biliodigestive, par laparotomie |
| HMGA001 | Ablation de calcul de la voie biliaire principale par cholédochotomie, par laparotomie |
| HMLA001 | Pose d'endoprothèse biliaire par cholédochotomie, par laparotomie |
| HNCA001 | Anastomose entre un faux kyste du pancréas et le duodénum [Kystoduodénostomie], par laparotomie |
| HNCA002 | Anastomose pancréaticojéjunale avec anastomose biliojéjunale, par laparotomie |
| HNCA003 | Anastomose pancréaticojéjunale avec anastomose biliojéjunale et gastrojéjunostomie, par laparotomie |
| HNCA004 | Anastomose pancréaticojéjunale avec gastrojéjunostomie, par laparotomie |
| HNCA005 | Anastomose entre un faux kyste du pancréas et le jéjunum [Kystojéjunostomie], par laparotomie |
| HNCA006 | Suture de plaie du pancréas avec reconstruction du conduit pancréatique, par laparotomie |
| HNCA007 | Anastomose pancréaticojéjunale, par laparotomie |
| HNCA008 | Anastomose entre un faux kyste du pancréas et l'estomac [Kystogastrostomie], par laparotomie |
| HNEA002 | Transplantation du pancréas et du rein, par laparotomie |
| HNEA900 | Transplantation du pancréas, par laparotomie |
| HNFA001 | Isthmectomie pancréatique avec rétablissement de continuité du conduit pancréatique, par laparotomie |
| HNFA002 | Pancréatectomie gauche avec conservation de la rate, avec anastomose pancréatojéjunale ou pancréaticojéjunale, par laparotomie |
| HNFA004 | Duodénopancréatectomie totale avec splénectomie [Splénopancréatectomie totale], par laparotomie |
| HNFA005 | Exérèse de tumeur du pancréas, par laparotomie |
| HNFA006 | Pancréatectomie totale ou subtotale avec conservation du duodénum et splénectomie, par laparotomie |
| HNFA007 | Duodénopancréatectomie céphalique, par laparotomie |
| HNFA008 | Pancréatectomie gauche avec conservation de la rate, par laparotomie |
| HNFA010 | Pancréatectomie gauche avec splénectomie [Splénopancréatectomie gauche] avec anastomose pancréatojéjunale ou pancréaticojéjunale, par laparotomie |
| HNFA011 | Pancréatectomie totale ou subtotale avec conservation du duodénum, sans splénectomie, par laparotomie |
| HNFA012 | Nécrosectomie pancréatique, par laparotomie |
| HNFA013 | Pancréatectomie gauche avec splénectomie [Splénopancréatectomie gauche], par laparotomie |
| HNJA001 | Drainage externe de collection pancréatique, par laparotomie |
| HPBA001 | Réduction de volume tumoral d'une maladie gélatineuse du péritoine, par laparotomie |
| HPCA001 | Dérivation péritonéoveineuse |
| HPFA003 | Exérèse de lésion d'un repli péritonéal [méso] sans résection intestinale, par laparotomie |
| HPFA004 | Résection du grand omentum [grand épiploon] [Omentectomie], par laparotomie |
| HPGA001 | Ablation d'un cathéter de dialyse péritonéale, par laparotomie |
| HPGA002 | Ablation d'un ballon intrapéritonéal, par laparotomie |
| HPKA002 | Changement d'un cathéter de dialyse péritonéale, par laparotomie |
| HPLA002 | Pose d'un ballon intrapéritonéal avant irradiation pelvienne, par laparotomie |
| HPNA001 | Destruction et/ou exérèse de lésion endométriosique superficielle du péritoine, par laparotomie |
| JACA001 | Suture de plaie ou de fracture du rein limitée au parenchyme, par abord direct |
| JACA002 | Néphrostomie cutanée, par abord direct |
| JADA001 | Néphropexie, par abord direct |
| JAEA002 | Autotransplantation du rein, par abord direct |
| JAEA003 | Transplantation du rein |
| JAFA001 | Héminéphrectomie avec urétérectomie partielle, par abord direct |
| JAFA002 | Néphrectomie totale, par lombotomie |
| JAFA003 | Héminéphrectomie avec urétérectomie totale, par abord direct |
| JAFA004 | Résection de kyste parasitaire du rein, par lombotomie |
| JAFA005 | Néphrectomie totale élargie à la loge rénale avec résection latérale de la veine cave inférieure, par abord direct |
| JAFA006 | Néphrectomie partielle ex situ avec autotransplantation du rein, par abord direct |
| JAFA007 | Résection de kyste non parasitaire du rein, par lombotomie |
| JAFA008 | Néphrectomie partielle avec dissection du pédicule vasculaire, par lombotomie ou par abord lombal postérieur |
| JAFA009 | Néphrectomie totale élargie à la loge rénale, par laparotomie ou par abord lomboabdominal |
| JAFA010 | Néphrectomie totale unilatérale, par abord lombal vertical |
| JAFA011 | Néphrectomie totale élargie à la loge rénale avec thrombectomie par cavotomie et contrôle intraatrial cardiaque, par thoraco-phréno-laparotomie |
| JAFA012 | Néphrectomie totale par laparotomie, avec extraction endoscopique rétrograde de l'uretère [stripping de l'uretère] |
| JAFA013 | Prélèvement d'un rein sur un donneur vivant, par abord direct |
| JAFA014 | Néphrectomie totale élargie à la loge rénale avec thrombectomie par cavotomie, par laparotomie ou par abord lomboabdominal |
| JAFA015 | Transplantectomie rénale, par abord direct |
| JAFA016 | Binéphrectomie, par laparotomie |
| JAFA019 | Néphrectomie partielle avec dissection du pédicule vasculaire, par laparotomie |
| JAFA021 | Néphrectomie totale élargie à la loge rénale avec surrénalectomie, par thoraco-phréno-laparotomie |
| JAFA022 | Néphrectomie totale élargie à la loge rénale avec thrombectomie par cavotomie, par thoraco-phréno-laparotomie |
| JAFA023 | Néphrectomie totale unilatérale, par laparotomie |
| JAFA024 | Néphrectomie partielle sans dissection du pédicule vasculaire, par lombotomie ou par abord lombal vertical |
| JAFA025 | Néphrectomie totale élargie à la loge rénale avec surrénalectomie et thrombectomie par cavotomie, par thoraco-phréno-laparotomie |
| JAFA026 | Héminéphrectomie pour fusion rénale, par abord direct |
| JAFA027 | Binéphrectomie, par abords lombaux verticaux |
| JAFA028 | Néphrectomie totale élargie à la loge rénale avec surrénalectomie et thrombectomie par cavotomie, par laparotomie ou par abord lomboabdominal |
| JAFA029 | Néphrectomie totale élargie à la loge rénale avec surrénalectomie, par laparotomie ou par abord lomboabdominal |
| JAFA030 | Néphrectomie partielle sans dissection du pédicule vasculaire, par laparotomie |
| JAFA031 | Néphrectomie totale élargie à la loge rénale, par thoraco-phréno-laparotomie |
| JAFA032 | Néphro-urétérectomie totale, par abord direct |
| JAGA001 | Ablation d'un calcul du rein par pyélotomie, par abord direct |
| JAGA002 | Ablation d'un calcul du rein par néphrotomie de la convexité, par abord direct |
| JAGA003 | Ablation de calculs multiples du rein par pyélotomie et/ou néphrotomie, par abord direct |
| JAGA004 | Ablation d'un calcul du rein par néphrotomie, par abord direct |
| JAGA005 | Ablation de calculs multiples du rein par néphrotomie de la convexité, par abord direct |
| JAGA006 | Ablation d'un calcul du rein par pyélotomie et néphrotomie, par abord direct |
| JAHA001 | Biopsie du rein, par abord direct |
| JAMA002 | Réparation d'un traumatisme complexe du rein et de la voie excrétrice, par abord direct |
| JAPA001 | Section de l'isthme d'un rein en fer à cheval, par abord direct |
| JASA001 | Hémostase de plaie ou de fracture du rein par pose de filet périrénal, par abord direct |
| JBCA001 | Pyélostomie cutanée, par abord direct |
| JBFA002 | Résection-anastomose du bassinet et de la jonction pyélo-urétérale, par abord direct |
| JBMA001 | Plastie du bassinet et de la jonction pyélo-urétérale, par abord direct |
| JCCA002 | Urétérostomie cutanée, par abord direct |
| JCCA003 | Suture de plaie ou de rupture de l'uretère, par abord direct |
| JCCA005 | Anastomose urétérocalicielle, par abord direct |
| JCCA006 | Urétérostomie cutanée transintestinale par anse non détubulée, par abord direct |
| JCCA007 | Dérivation urinaire par anastomose urétéro-urétérale homolatérale, par abord direct |
| JCCA008 | Dérivation urinaire par anastomose urétéro-urétérale croisée, par abord direct |
| JCCA009 | Dérivation de l'urine par conduit rénovésical prothétique souscutané, avec néphrostomie |
| JCCA010 | Dérivation de l'urine par conduit rénovésical prothétique souscutané, sans néphrostomie |
| JCCA011 | Urétérostomie cutanée transintestinale avec création d'un réservoir continent, par abord direct |
| JCCA012 | Anastomose urétérocolique ou urétérorectale avec confection d'un réservoir détubulé rectosigmoïdien ou iléo-recto-sigmoïdien, par abord direct |
| JCCA013 | Anastomose urétérocolique ou urétérorectale directe, par abord direct |
| JCCA014 | Urétérostomie cutanée avec anastomose urétéro-urétérale croisée, par abord direct |
| JCEA001 | Réimplantation urétérovésicale bilatérale avec création de montage antireflux, par abord direct |
| JCEA002 | Réimplantation urétérovésicale unilatérale avec création de montage antireflux, par abord direct |
| JCEA003 | Réimplantation urétérovésicale avec plastie de la vessie par mobilisation [vessie psoïque] ou allongement par lambeau pédiculé, par abord direct |
| JCEA004 | Réimplantation vésicale d'une duplicité urétérale, par abord direct |
| JCEA005 | Réimplantation urétérovésicale sans création de montage antireflux, par abord direct |
| JCFA001 | Urétérectomie totale, par abord direct |
| JCFA002 | Urétérectomie segmentaire lombo-iliaque avec rétablissement de la continuité de l'uretère, par abord direct |
| JCFA003 | Urétérectomie segmentaire pelvienne avec rétablissement de la continuité de l'uretère, par abord direct |
| JCFA004 | Exérèse d'une urétérocèle avec réimplantation urétérovésicale, héminéphrectomie et urétérectomie totale homolatérales, par abord direct |
| JCFA005 | Résection longitudinale modelante d’un méga-uretère avec réimplantation urétérovésicale et montage antireflux, par abord direct |
| JCFA006 | Exérèse unilatérale d'une urétérocèle avec réimplantation urétérovésicale bilatérale, par abord direct |
| JCFA007 | Exérèse d'une urétérocèle avec réimplantation urétérovésicale homolatérale, par abord direct |
| JCFA008 | Urétérectomie segmentaire pelvienne avec réimplantation urétérovésicale et montage antireflux, par abord direct |
| JCFA009 | Urétérectomie segmentaire pelvienne avec réimplantation urétérovésicale, par abord direct |
| JCFA010 | Urétérectomie segmentaire pelvienne avec réimplantation urétérovésicale et plastie de la vessie par mobilisation [vessie psoïque] ou allongement par lambeau pédiculé, par abord direct |
| JCGA001 | Ablation de calcul de l'uretère pelvien, par abord direct |
| JCGA002 | Ablation de calcul de l'uretère lombal, par abord direct |
| JCGA003 | Ablation de calcul de l'uretère iliaque, par abord direct |
| JCKA001 | Remplacement partiel de l'uretère par un segment digestif, par abord direct |
| JCKA002 | Remplacement total de l'uretère par un segment digestif, par abord direct |
| JCMA001 | Transformation d'une urétérostomie cutanée en anastomose urétérocolique directe |
| JCMA002 | Transformation d'une urétérostomie cutanée en dérivation interne avec confection d'un réservoir détubulé rectosigmoïdien ou iléo-recto-sigmoïdien |
| JCMA003 | Transformation d'une urétérostomie cutanée en dérivation interne par réimplantation urétérovésicale |
| JCMA004 | Création d'un montage urétérovésical antireflux sans réimplantation urétérovésicale, par abord direct |
| JCMA005 | Transformation d'une urétérostomie cutanée en dérivation interne avec entérocystoplastie de remplacement [néovessie] orthotopique par anse intestinale détubulée |
| JCPA001 | Libération de l'uretère avec intrapéritonisation ou lambeau péritonéal, par abord direct |
| JCPA002 | Libération de l'uretère sans intrapéritonisation, par abord direct |
| JCPA003 | Section-anastomose d'un uretère rétrocave, par abord direct |
| JCSA001 | Fermeture de fistule urétérodigestive acquise, par abord direct |
| JCSA002 | Fermeture de fistule urétéro-utérine acquise, par abord direct |
| JCSA003 | Fermeture de fistule urétérocutanée acquise, par abord direct |
| JCSA004 | Fermeture de fistule urétérovaginale acquise, par abord direct |
| JCSA005 | Fermeture de fistule urétérovésicale acquise, par abord direct |
| JDFA001 | Cystectomie totale avec urétérostomie cutanée, par laparotomie |
| JDFA003 | Cystectomie totale avec urétérostomie cutanée transintestinale par anse détubulée continente, par laparotomie |
| JDFA004 | Cystectomie supratrigonale avec entérocystoplastie détubulée d'agrandissement, par laparotomie |
| JDFA005 | Cystectomie totale, par laparotomie |
| JDFA006 | Cystectomie totale avec anastomose urétérocolique et confection d'un réservoir détubulé rectosigmoïdien ou iléo-recto-sigmoïdien, par laparotomie |
| JDFA008 | Cystectomie totale avec urétérostomie cutanée transintestinale par anse non détubulée, par laparotomie |
| JDFA009 | Cystectomie totale avec anastomose urétérocolique directe, par laparotomie |
| JDFA010 | Exérèse de la plaque d'une exstrophie vésicale avec dérivation urinaire, avec ostéotomie du bassin |
| JDFA012 | Exérèse de la plaque d'une exstrophie vésicale avec dérivation urinaire, sans ostéotomie du bassin |
| JDFA013 | Exérèse de la plaque d'une exstrophie vésicale avec dérivation urinaire et cure unilatérale ou bilatérale de hernie de l'aine, sans ostéotomie du bassin |
| JDFA014 | Cystectomie partielle avec implantation de matériel pour irradiation interstitielle de la vessie, par laparotomie |
| JDFA015 | Cystectomie supratrigonale avec entérocystoplastie détubulée d'agrandissement et réimplantation urétérovésicale, par laparotomie |
| JDFA016 | Cystectomie totale avec entérocystoplastie de remplacement [néovessie] orthotopique par anse détubulée, par laparotomie |
| JDFA017 | Cystectomie partielle avec réimplantation urétérovésicale, par laparotomie |
| JDFA019 | Vésiculo-prostato-cystectomie totale avec anastomose urétérocolique directe, par laparotomie |
| JDFA020 | Vésiculo-prostato-cystectomie totale avec urétérostomie cutanée transintestinale par anse détubulée continente, par laparotomie |
| JDFA021 | Vésiculo-prostato-cystectomie totale avec entérocystoplastie de remplacement [néovessie] orthotopique par anse détubulée, par laparotomie |
| JDFA022 | Vésiculo-prostato-cystectomie totale avec anastomose urétérocolique et confection d'un réservoir détubulé rectosigmoïdien ou iléo-recto-sigmoïdien, par laparotomie |
| JDFA023 | Vésiculo-prostato-cystectomie totale avec urétérostomie cutanée, par laparotomie |
| JDFA024 | Vésiculo-prostato-cystectomie totale, par laparotomie |
| JDFA025 | Vésiculo-prostato-cystectomie totale avec urétérostomie cutanée transintestinale par anse non détubulée, par laparotomie |
| JDLA001 | Implantation vésicale de vecteur pour curiethérapie interstitielle de la vessie, par laparotomie |
| JDMA002 | Entérocystoplastie d'agrandissement avec réimplantation urétérovésicale bilatérale, par laparotomie |
| JDSA001 | Fermeture d'une exstrophie vésicale avec allongement de l'urètre chez la fille, avec ostéotomie du bassin |
| JDSA003 | Fermeture d'une exstrophie vésicale avec allongement du pénis, sans ostéotomie du bassin |
| JDSA004 | Fermeture d'une exstrophie vésicale avec allongement de l'urètre chez la fille, sans ostéotomie du bassin |
| JDSA007 | Fermeture d'une exstrophie vésicale avec allongement du pénis et cure unilatérale ou bilatérale de hernie de l'aine, sans ostéotomie du bassin |
| JDSA009 | Fermeture d'une exstrophie vésicale avec allongement du pénis, avec ostéotomie du bassin |
| JDSA011 | Fermeture de fistule vésicodigestive acquise, par laparotomie |
| JEMA018 | Urétrocervicoplastie de continence avec réimplantation urétérovésicale et création de montage antireflux, par laparotomie |
| JFFA001 | Pelvectomie antérieure avec urétérostomie cutanée, par laparotomie |
| JFFA002 | Pelvectomie postérieure avec rétablissement de la continuité digestive, par laparotomie |
| JFFA003 | Pelvectomie antérieure avec urétérostomie cutanée transintestinale par anse non détubulée, par laparotomie |
| JFFA004 | Pelvectomie postérieure sans rétablissement de la continuité digestive, par laparotomie et par abord périnéal |
| JFFA005 | Pelvectomie postérieure sans rétablissement de la continuité digestive, par laparotomie |
| JFFA006 | Exérèse de lésion de l'espace rétropéritonéal sans dissection des gros vaisseaux, par thoraco-phréno-laparotomie |
| JFFA007 | Exérèse d'une lipomatose pelvienne, par laparotomie |
| JFFA008 | Pelvectomie totale avec urétérostomie cutanée, par laparotomie et par abord périnéal |
| JFFA008 | Pelvectomie totale avec urétérostomie cutanée, par laparotomie et par abord périnéal |
| JFFA009 | Pelvectomie antérieure avec anastomose urétérocolique directe, par laparotomie |
| JFFA010 | Exérèse de lésion de l'espace rétropéritonéal sans dissection des gros vaisseaux, par laparotomie ou par lombotomie |
| JFFA011 | Pelvectomie totale avec urétérostomie cutanée transintestinale par anse non détubulée, par laparotomie et par abord périnéal |
| JFFA011 | Pelvectomie totale avec urétérostomie cutanée transintestinale par anse non détubulée, par laparotomie et par abord périnéal |
| JFFA013 | Pelvectomie totale avec urétérostomie cutanée transintestinale par anse détubulée continente, par laparotomie et par abord périnéal |
| JFFA013 | Pelvectomie totale avec urétérostomie cutanée transintestinale par anse détubulée continente, par laparotomie et par abord périnéal |
| JFFA014 | Exérèse de lésion endométriosique de la cloison rectovaginale, par laparotomie |
| JFFA016 | Pelvectomie antérieure avec urétérostomie cutanée transintestinale par anse détubulée continente, par laparotomie |
| JFFA018 | Pelvectomie antérieure, par laparotomie |
| JFFA019 | Pelvectomie antérieure avec anastomose urétérocolique et confection d'un réservoir détubulé rectosigmoïdien ou iléo-recto-sigmoïdien, par laparotomie |
| JFFA021 | Exérèse de lésion de l'espace rétropéritonéal avec dissection des gros vaisseaux, par abord direct |
| JFFA022 | Pelvectomie antérieure avec entérocystoplastie de remplacement [néovessie] orthotopique par anse détubulée, par laparotomie |
| JFJA001 | Évacuation de collection périrénale, par abord direct |
| JJFA006 | Exérèse de reliquat embryonnaire du ligament large, par laparotomie |
| LLFA003 | Exérèse partielle du diaphragme sans pose de prothèse, par thoracotomie |
| LLFA013 | Exérèse partielle du diaphragme avec pose de prothèse, par thoracotomie |
| LLMA001 | Cure d'une hernie acquise de la coupole gauche du diaphragme, par thoracotomie |
| LLMA002 | Cure d'une hernie hiatale congénitale avec agénésie des piliers du diaphragme, par laparotomie |
| LLMA004 | Plastie musculaire ou prothétique d'une coupole du diaphragme pour hernie congénitale, par laparotomie |
| LLMA005 | Cure d'une hernie acquise de la coupole droite du diaphragme, par abord direct |
| LLMA006 | Cure d'une hernie hiatale sans pose de prothèse, par laparotomie |
| LLMA007 | Cure d'une hernie rétrocostoxiphoïdienne, par laparotomie |
| LLMA009 | Plastie musculaire ou prothétique d'une coupole du diaphragme pour hernie congénitale, par thoracotomie |
| LLMA010 | Cure d'une hernie hiatale avec pose de prothèse, par laparotomie |
| LMFA001 | Exérèse de tumeur de la paroi abdominale antérieure avec fermeture par suture, par abord direct |
| LMFA002 | Exérèse de tumeur de la paroi abdominale antérieure avec fermeture par prothèse, par abord direct |
| LMMA005 | Réparation de perte de substance transfixiante de la paroi abdominale par lambeau libre et prothèse |
| ZCJA002 | Évacuation d'une collection intraabdominale, par laparotomie |
| ZCJA003 | Évacuation de collection intraabdominale, par colpotomie |
| ZCJA004 | Évacuation de plusieurs collections intraabdominales, par laparotomie |
| ZCJA005 | Évacuation de collection intraabdominale, par thoracotomie |

| Supplementary table 1b. List of ICD-10 codes used for sepsis diagnosis | |
| --- | --- |
|  |  |
| R65.0 | Syndrome de réponse inflammatoire systémique (SRIS) d’origine infectieuse sans défaillance d’organe |
| R65.1 | Syndrome de réponse inflammatoire systémique (SRIS) d’origine infectieuse avec défaillance d’organe |
| R57.2 | Choc septique |
| A20.7 | Peste septicémique |
| A22.7 | Sepsis charbonneux |
| A24.1 | Mélioïdose aiguë et galopante |
| A26.7 | Sepsis à Erysipelothrix |
| A32.7 | Sepsis Listérien |
| A39.2 | Méningococcémie aiguë |
| A39.3 | Méningococcémie chronique |
| A39.4 | Méningococcémie, sans précision *Bactériémie méningococcique SAI* |
| A42.7 | Sepsis actinomycosique |
| A48.3 | Syndrome du choc toxique |
| A21.7 | Tularémie généralisée |
| A40* | Sepsis à streptocoques |
| A41* | Autres sepsis |
| A49.9 | Infection bactérienne, sans precision *Bactériémie SAI* |
| B37.7 | Sepsis à Candida |
| B00.7 | Maladie disséminée due au virus de l'herpès *Sepsis dû au virus de l'herpès* |
| O75.3 | Autres infections au cours du travail *Sepsis au cours du travail* |
| O85 | Sepsis puerpéral |
| P36* | Sepsis du nouveau-né |
| T80.2 | Infections consécutives à une injection thérapeutique, une perfusion et une transfusion *Sepsis consécutif à injection thérapeutique, perfusion et transfusion* |
| T88.0 | Infection consécutive à vaccination *Sepsis consécutif à vaccination* |
|  |  |

| Supplementary table 1c. List of ICD-10 and CCAM codes used for cardiac failure, diabetes, chronic kidney disease diagnoses | |
| --- | --- |
| I50* | Insuffisance cardiaque |
| I11.0 | Cardiopathie hypertensive, avec insuffisance cardiaque (congestive) |
| I13.0 | Cardionéphropathie hypertensive, avec insuffisance cardiaque (congestive) |
| I13.2 | Cardionéphropathie hypertensive, avec insuffisance cardiaque (congestive) et rénale |
| I13.9 | Cardionéphropathie hypertensive, sans précision |
| K76.1 | Congestion passive chronique du foie |
| E10* | Diabète sucré de type 1 |
| E11* | Diabète sucré de type 2 |
| E12* | Diabète sucré de malnutrition |
| E13* | Autres diabètes sucrés précisés |
| E14* | Diabète sucré, sans précision |
| G59.0* | Mononévrite diabétique |
| G63.2* | Polynévrite diabétique |
| G73.0* | Syndrome myasthénique au cours de maladies endocriniennes |
| H28.0* | Cataracte diabétique |
| H36.0* | Rétinopathie diabétique |
| M14.2* | Arthropathie diabétique |
| N08.3* | Glomérulopathie au cours du diabète sucré |
| N18* | Insuffisance rénale chronique |
| Z99.2 | Dépendance envers une dialyse rénale |
| JVJB001 | Séance d'épuration extrarénale par dialyse péritonéale pour insuffisance rénale chronique |
| JVJF004 | Séance d'épuration extrarénale par hémodialyse pour insuffisance rénale chronique |
| JVJF008 | Séance d'épuration extrarénale par hémodiafiltration, hémofiltration ou biofiltration sans acétate pour insuffisance rénale chronique |
| JVRP007 | Séance d'entraînement à la dialyse péritonéale automatisée |
| JVRP008 | Séance d'entraînement à la dialyse péritonéale continue ambulatoire |
| JVRP004 | Séance d'entraînement à l'hémodialyse |

| Supplementary table 2. Characteristics of the population by subgroup | | | | | | | | | |
| --- | --- | --- | --- | --- | --- | --- | --- | --- | --- |
|  | **Cardiovascular surgery with ECC** | | | **Major open visceral surgery** | | | **Sepsis** | | |
|  | AKI | No AKI | All | AKI | No AKI | All | AKI | No AKI | All |
|  | n=7,165 | n=32,919 | n=40,084 | n=15,387 | n=151,444 | n=166,831 | n=83,553 | n=337,990 | n=421,543 |
| Hospital status |  |  |  |  |  |  |  |  |  |
| -Private | 1,312 (18.3) | 10,615 (32.2) | 11,927 (29.8) | 2,247 (14.6) | 57,719 (38.1) | 59,966 (35.9) | 6,837 (8.2) | 50,203 (14.9) | 57,040 (13.5) |
| -Public | 5,853 (81.7) | 22,304 (67.8) | 28,157 (70.2) | 13,140 (85.4) | 93,725 (61.9) | 106,865 (64.1) | 76,716 (91.8) | 287,787 (85.1) | 364,503 (86.5) |
| Risk factors for AKI |  |  |  |  |  |  |  |  |  |
| -Sepsis | 1,792 (25.0) | 1,924 (5.8) | 3,716 (9.3) | 7,774 (50.5) | 16,710 (11.0) | 24,484 (14.7) | - | - | - |
| -Surgery | - | - | - | - | - | - | 25,328 (30.3) | 84,125 (24.9) | 109,453 (26.0) |
| -Heart failure | 2,979 (41.6) | 6,505 (19.8) | 9,484 (23.7) | 1,765 (11.5) | 2,829 (1.9) | 4,594 (2.8) | 20,185 (24.2) | 32,308 (9.6) | 52,493 (12.5) |
| -Diabetes | 1,840 (25.7) | 6,888 (20.9) | 8,728 (21.8) | 3,544 (23.0) | 17,399 (11.5) | 20,943 (12.6) | 22,896 (27.4) | 53,952 (16.0) | 76,848 (18.2) |
| -Chronic kidney disease | 1,415 (19.7) | 1,275 (3.9) | 2,690 (6.7) | 3,492 (22.7) | 7,464 (4.9) | 10,956 (6.6) | 18 553 (22.2) | 25,742 (7.6) | 44,295 (10.5) |
| Treatments during hospital stay |  |  |  |  |  |  |  |  |  |
| -Vasopressors | 5,498 (76.7) | 14,874 (45.2) | 20,372 (50.8) | 7,712 (50.1) | 8,138 (5.4) | 15,850 (9.5) | 29 250 (35.0) | 22 444 (6.6) | 51 694 (12.3) |
| -MV | 6,051 (84.5) | 24,863 (75.5) | 30,914 (77.1) | 7,598 (49.4) | 9,586 (6.3) | 17,184 (10.3) | 24 403 (29.2) | 22 377 (6.6) | 46 780 (11.1) |
| -Days under MV, mean (SD), days | 6.5 (13.4) | 1.7 (3.1) | 2.6 (6.8) | 10.2 (22.9) | 4.7 (8.8) | 7.1 (16.8) | 11,5 (19.1) | 9.1 (13.7) | 10,4 (16.8) |
| - RRT for AKI | 2,121 (29.6) | 0 (0.0) | 2,121 (5.3) | 4,013 (26.1) | 0 (0.0) | 4,013 (2.4) | 14 522 (17.4) | 0 (0.0) | 14 522 (3.4) |
| Admission |  |  |  |  |  |  |  |  |  |
| -From home | 5,152 (71.9) | 27,619 (83.9) | 32,771 (81.8) | 8,277 (53.8) | 120,030 (79.3) | 128,307 (76.9) | 21,536 (25.8) | 109,680 (32.5) | 131,216 (31.1) |
| -From MSO | 1,058 (14.8) | 3,373 (10.2) | 4,431 (11,1) | 1,285 (8.4) | 4,445 (2.9) | 5,730 (3.4) | 10,835 (13.0) | 30,607(9.1) | 41,442 (9.8) |
| -From ER | 833 (11.6) | 1,548 (4.7) | 2,381 (5.9) | 5,605 (36.4) | 25,561 (16.9) | 31,166 (18.7) | 48,945 (58.6) | 188,976 (55.9) | 237,921 (56.4) |
| Discharge |  |  |  |  |  |  |  |  |  |
| -To home | 1,663 (23.2) | 10 433 (31.7) | 12,096 (30.2) | 7,077 (46.0) | 124,858 (82.4) | 131,935 (79.1) | 32,413 (38.8) | 219,657 (65.0) | 252,070 (59.8) |
| -To MSO | 2,066 (28.8) | 8,704 (26.4) | 10,770 (26.9) | 1,897 (12.3) | 7,791 (5.1) | 9,688 (5.8) | 12,665 (15.2) | 39,580 (11.7) | 52,245 (12.4) |
| -To rehabilitation  care | 2,151 (30.0) | 12,974 (39.4) | 15,125 (37.7) | 2,187 (14.2) | 13,085 (8.6) | 15,272 (9.2) | 10,687 (12.8) | 32,017 (9.5) | 42,704 (10.1) |
| Death | 1,196 (16.7) | 542 (1.6) | 1,738 (4.3) | 3,870 (25.2) | 3,683 (2.4) | 7,553 (4.5) | 24,672 (29.5) | 34,786 (10.3) | 59,458 (14.1) |
| ICU stay (yes) | 6,883 (96.1) | 29,215 (88.7) | 36,098 (90.1) | 9,143 (59.4) | 14,207 (9.4) | 23,350 (14.0) | 34,688 (41.5) | 35,962 (10.6) | 70,550 (16.7) |
| Patient characteristics |  |  |  |  |  |  |  |  |  |
| -Age, mean (SD), years | 64.8 (17.2) | 61.5 (19.0) | 62.1 (18.8) | 67.1 (16.7) | 62.1 (18.6) | 62.5 (18.5) | 72.1 (16.1) | 60.3 (26.6) | 62.7 (25.3) |
| -Male sex | 5,163 (72.1) | 23,620 (71.8) | 28,783 (71.8) | 9,742 (63.3) | 77,672 (51.3) | 87,414 (52.4) | 50,059 (59.9) | 183,145 (54.2) | 233,204 (55.3) |
| Data are presented as number and percentages (%) unless otherwise specified.  MSO = medical surgical and obstetrics units, ER = emergency room, MV = mechanical ventilation, ECC = extracoroporeal circulation, AKI = Acute Kidney injury, RRT = renal replacement therapy, ICU= intensive care unit | | | | | | | | | |
